# TLR3 agonist and Sorafenib combinatorial therapy promotes immune activation and controls hepatocellular carcinoma progression

**DOI:** 10.18632/oncotarget.4583

**Published:** 2015-07-23

**Authors:** Victor Ho, Tong Seng Lim, Justin Lee, Jeffrey Steinberg, Radoslaw Szmyd, Muly Tham, Jadegoud Yaligar, Philipp Kaldis, Jean-Pierre Abastado, Valerie Chew

**Affiliations:** ^1^ Singapore Immunology Network (SIgN), Agency for Science, Technology and Research (A*STAR), Biopolis, Singapore; ^2^ Singhealth Translational Immunology and Inflammation Centre (STIIC), Singapore Health Services Pte Ltd, Singapore; ^3^ Singapore Bioimaging Consortium (SBIC), Agency for Science, Technology and Research (A*STAR), Biopolis, Singapore; ^4^ Institute Molecular and Cell Biology (IMCB), Agency for Science, Technology and Research (A*STAR), Biopolis, Singapore; ^5^ Department of Biochemistry, National University of Singapore (NUS), Singapore; ^6^ Institut de Recherches Internationales Servier, Suresnes, France

**Keywords:** cancer immunotherapy, tumor microenvironment, local immune activation, hepatocellular carcinoma, combinatorial treatment

## Abstract

Hepatocellular carcinoma (HCC) is associated with high mortality and the current therapy for advanced HCC, Sorafenib, offers limited survival benefits. Here we assessed whether combining the TLR3 agonist: lysine-stabilized polyinosinic-polycytidylic-acid (poly-ICLC) with Sorafenib could enhance tumor control in HCC. Combinatorial therapy with poly-ICLC and Sorafenib increased apoptosis and reduced proliferation of HCC cell lines *in vitro*, in association with impaired phosphorylation of AKT, MEK and ERK. *In vivo*, the combinatorial treatment enhanced control of tumor growth in two mouse models: one transplanted with Hepa 1-6 cells, and the other with liver tumors induced using the *Sleeping beauty* transposon. Tumor cell apoptosis and host immune responses in the tumor microenvironment were enhanced. Particularly, the activation of local NK cells, T cells, macrophages and dendritic cells was enhanced. Decreased expression of the inhibitory signaling molecules PD-1 and PD-L1 was observed in tumor-infiltrating CD8^+^ T cells and tumor cells, respectively. Tumor infiltration by monocytic-myeloid derived suppressor cells (Mo-MDSC) was also reduced indicating the reversion of the immunosuppressive tumor microenvironment. Our data demonstrated that the combinatorial therapy with poly-ICLC and Sorafenib enhances tumor control and local immune response hence providing a rationale for future clinical studies.

## INTRODUCTION

Hepatocellular carcinoma (HCC) is the sixth most common cancer and the second most frequent cause of cancer-related death worldwide [1]. The first-line treatment for HCC is surgical resection or liver transplantation which are restricted to patients with early disease and the availability of suitable donor organs. At present, the only FDA-approved treatment for advanced HCC is the multikinase inhibitor-Sorafenib, which confers only limited improvement in patient survival (~4 months) and exhibits a poor response rate of just ~2% [2]. Recent studies have also reported the emergence of HCC resistance to Sorafenib treatment that is associated with enhanced metastatic potential of tumor cells and subsequent relapse after prolonged treatment [3, 4]. There is hence an urgent need for more efficient therapies for HCC.

Sorafenib exerts anti-proliferative and anti-angiogenesis effects by disrupting signaling through the vascular endothelial growth factor receptor, platelet-derived growth factor receptor, and RAF/MEK/ERK pathway [5]. Several studies have shown that Sorafenib induces host immunosuppression by impairing the activation of natural killer (NK) cells and dendritic cells (DC) [6, 7]. Other studies have indicated that Sorafenib is able to reduce the proportion of PD-1-expressing CD8^+^ T cells and decreases the number and function of regulatory T cells (Treg) in the tumor microenvironment [8]. Since the balance of immune responses in the tumor microenvironment is known to be a critical determinant of tumor progression, it will be important to better understand the immuno-modulatory effects of Sorafenib and to determine if methods of promoting host immune response could increase its efficacy.

We have previously reported that a pro-inflammatory microenvironment including the expression of Toll-like receptor (TLR)3 is associated with superior patient survival in HCC [9, 10]. Accordingly, we have demonstrated that TLR3 triggering can directly induce the apoptosis of TLR3-expressing tumor cells, and the expression of chemokine CXCL10 and CCL5 which promote infiltration of T cells and NK cells into the tumor [11]. Indeed, TLR3 agonists-induced tumor cell death was reported in multiple cancer cell lines as well [12–14]. In our previous study, we further demonstrated TLR3 agonists-induced activation of tumor-infiltrating NK cells and T cells leading to better tumor control [11]. Consistent with these data, recent testing of TLR3 agonists in treating various cancers has generated promising results and displayed limited toxicity in both mouse models and clinical trials [15].

It is increasingly recognized that a combinatorial approach to cancer treatment could be more effective than monotherapy, particularly in cancers such as HCC that currently lack effective treatment options [16–19]. In the current study, we assessed the use of the GMP-grade TLR3 agonist poly-ICLC (lysine-stabilized poly-IC, or Hiltonol™) in combination with Sorafenib for the treatment of liver tumors. Our data suggest that combining poly-ICLC with Sorafenib can significantly reduce tumor growth both *in vitro* and *in vivo* by direct impairment of tumor cell survival and proliferation as well as potent activation of host immune responses within the tumor microenvironment.

## RESULTS

### Poly-ICLC treatment enhances tumor control in mice

**Figure 1:**
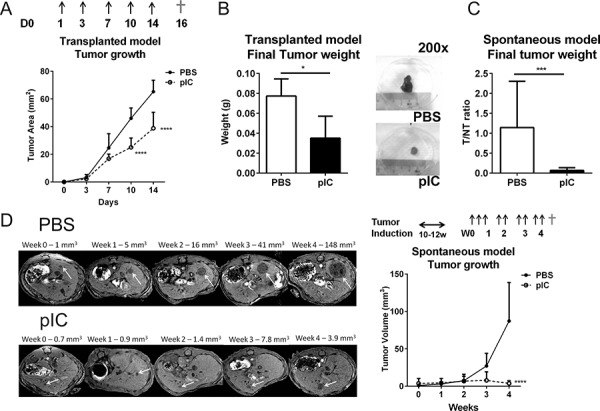
Poly-ICLC restricts tumor growth in murine models of liver tumors **A&B.** C57BL/6 mice transplanted with Hepa 1-6 cells were treated with PBS or poly-ICLC (pIC) on the indicated days (arrows). *n* = 5 each group. A. Slowed tumor growth indicated as reduced tumor areas (mm^2^) in mice treated with pIC versus PBS on d10: 25.0 ± 6.7 vs. 46.0 ± 7.5; *p* < 0.0001 and on d14: 38.8 ± 11.6 vs.65.2 ± 8.3; *p* < 0.0001. B. Left, Reduced final tumor weights (g) on d16 (†) in pIC- versus PBS-treated mice: 0.035 ± 0.022 vs.0.077 ± 0.017; *p* = 0.03. Right, representative images of tumors harvested from treated mice. Diameter of 6-well plate = 38 mm. **C&D.** C57BL/6mice were induced to develop spontaneous liver tumors and administered with PBS or pIC as indicated (arrows). *n* = 8 each group. C. Decreased mass ratio of liver tumor to non-tumorous liver tissue as harvested at week-4(†) from pIC- versus PBS-treated mice: 0.065 ± 0.069 vs.1.142 ± 1.161; *p* = 0.0006. D. Representative MRI scanning images of livers (left) and tumor volumes measured from these images (Right) showing slowed tumor growth and reduced tumor volume (mm^3^) in mice treated with pIC versus PBS: 3.7 ± 3.5 vs.87.1 ± 51.6; *p* < 0.0001. For all graphs, mean and SD are shown. *p < 0.05, ***p < 0.001, ****p < 0.0001, A&D. two-way ANOVA with Sidak's multiple comparisons test. B&C. Mann-Whitney Test.

We have previously shown that the TLR3 agonists polyinosinic:polycytidylic acid (poly-IC) and polyadenylic-polyuridylic acid (poly-AU) promote control of tumor growth in the murine models of liver tumor [11]. Here, we extended our studies to assess whether monotherapy with the GMP-grade TLR3 agonist poly-ICLC, could restrict tumor growth in both transplanted and spontaneous models of liver tumors. In mice transplanted with Hepa 1-6 cells, treatment with poly-ICLC (pIC) led to a significant reduction in tumor growth compared with PBS-treated controls, as shown by tumor area measurement on d10 and d14 (Figure 1A). The final harvested tumor weight was also significantly reduced in pIC-treated mice (Figure 1B). We then assessed whether this beneficial effect of pIC treatment could be replicated in another mouse model in which liver tumors were induced 10–12 weeks after hydrodynamic tail-vein injection of a cocktail comprising oncogenes NRas and shRNAp53 and SB13 transposase. pIC treatment in these mice lead to significant reduction in mass ratio of liver tumor to non-tumourous liver tissue (Figure 1C). The tumor volume compared with PBS-treated controls as assessed by weekly magnetic resonance imaging (MRI) was also significantly lower in pIC-treated mice (Figure 1D). These data were consistent with our previous report showing that liver tumor growth can be restricted by specific TLR3 agonists [11].

### Combinatorial treatment with poly-ICLC and Sorafenib enhances control of tumor growth as compared to monotherapy

**Figure 2:**
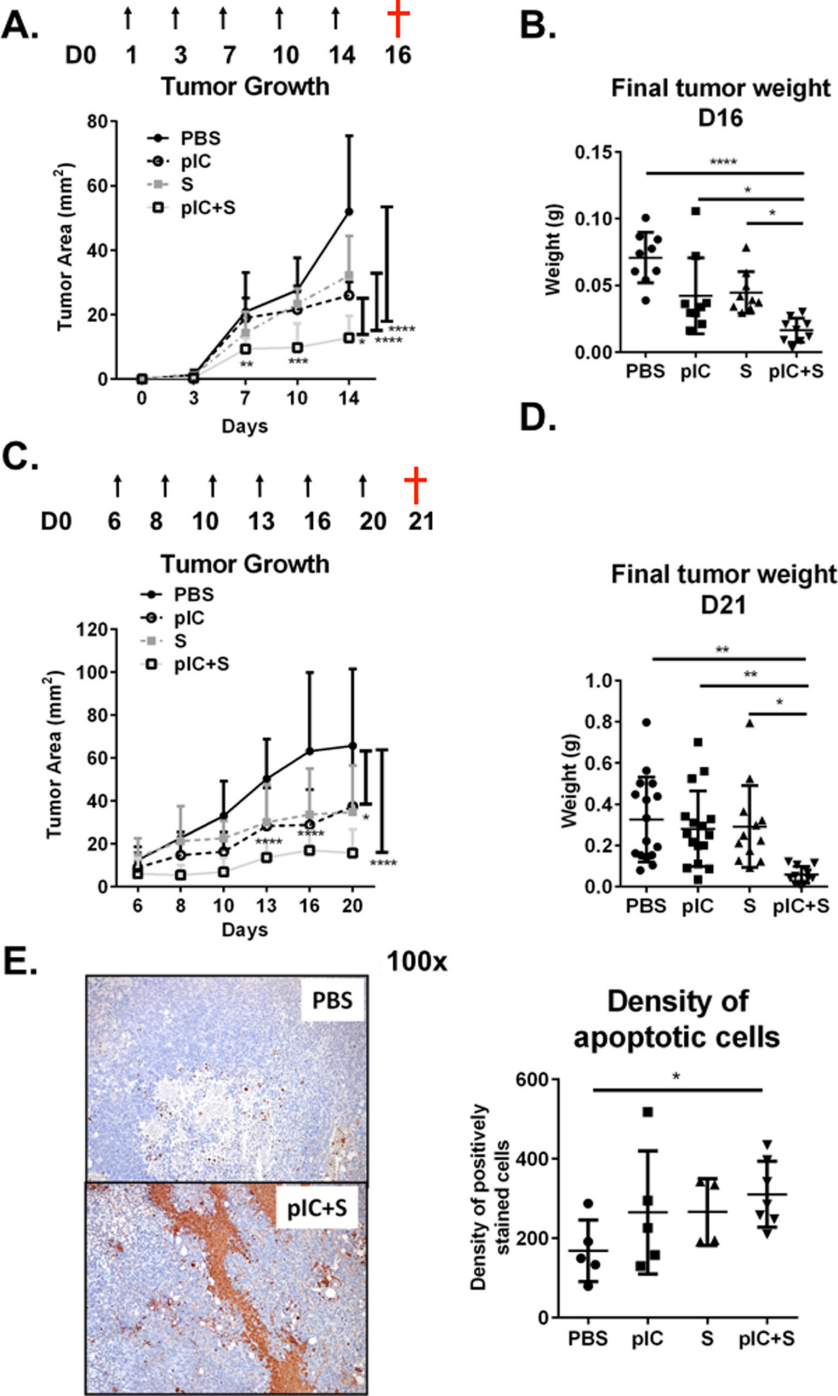
Combination of poly-ICLC and Sorafenib enhanced tumor control in mice C57BL/6 mice were transplanted with Hepa 1-6 cells and PBS or poly-ICLC (pIC) were given on the indicated days (arrows) and Sorafenib (S) was included in the diet. **A&B.** Treatment started on day 1. *n* = 8–9 each group. **A.** Slowed tumor growth on d14, pIC+S: 12.8 ± 6.8 mm^2^ vs. PBS: 52.0 ± 23.5 mm^2^, *p* < 0.0001; vs. pIC: 26.0 ± 4.2 mm^2^, *p* = 0.011 and vs. S: 32.2 ± 12.2 mm^2^, *p* < 0.0001 and **B.** Decreased final tumor weights (g) as harvested on day 16 (†) in pIC+S-treated mice: 0.016 ± 0.009 vs. PBS: 0.071 ± 0.019, *p* < 0.0001; vs. pIC: 0.042 ± 0.029, *p* = 0.038 and vs. S: 0.045 ± 0.016, *p* = 0.021. **C&D.** Tumors were allowed to grow until day 6 before treatments were given. *n* = 12–16 each group. **C.** Slowed tumor growth (mm^2^) on day 20, pIC+S: 15.6 ± 11.1 vs. PBS: 65.6 ± 35.8, *p* < 0.0001; vs. pIC: 37.3 ± 28.5, *p* = 0.011 and vs. S: 34.7 ± 21.8, *p* = 0.029 and **D.** decreased final tumor weights (g) as harvested on day 21 (†) in pIC+S-treated mice: 0.058 ± 0.039 vs. PBS: 0.326 ± 0.206, *p* = 0.011; vs. pIC: 0.281 ± 0.183, *p* = 0.0083; and vs. S: 0.292 ± 0.199, *p* = 0.0101. **E.** Left, representative immunohistochemistry images of apoptotic cells (brown) as identified by TUNEL-assay (100× magnification, scale bar = 20 μm). Right, Increased density of apoptotic cells per tumor field from mice treated with pIC+S: 311 ± 83 vs. PBS: 168 ± 78; *p* = 0.028. Data represent *n* = 4–7 from each group. For all graphs, mean and SD are shown. *p < 0.05; **p < 0.01, ***p < 0.001, ****p < 0.0001. A&C. Two-way ANOVA with Tukey's multiple comparisons test. B, D & E. One-way ANOVA test with post Tukey's multiple comparisons test. Points represent individual tumors.

Sorafenib is currently the only FDA-approved drug available for advanced HCC but confers only limited survival benefit in patients [2]. Since we observed that poly-ICLC administration promoted control of tumor growth in our HCC models, we next aimed to examine whether combining poly-ICLC with Sorafenib could further decrease tumor burden/growth in mouse models of liver tumors. C57BL/6 mice transplanted with Hepa 1-6 cells were administered with PBS, poly-ICLC (pIC), Sorafenib (S), or in combination (pIC+S). We observed that tumor area was significantly reduced by co-treatment when compared with monotherapy or PBS-treated controls (Figure 2A). Final tumor mass was similarly reduced (Figure 2B). We therefore sought to determine whether the effects of this combinatorial therapy would extend to well-established tumors that were allowed to grow to an average area of 10 mm^2^ over 6 days before treatment. Even under these conditions, co-treatment with poly-ICLC and Sorafenib was able to significantly restrict tumor growth compared with monotherapy or PBS-treated controls (Figure 2C). Final tumor mass was again significantly reduced (Figure 2D). Consistent with these data, we observed significant increase in apoptotic tumor cells in animals that received combinatorial treatment (Figure 2E). An initial loss of body weight was noted in mice that were treated with either poly-ICLC or combinatorial therapy, but this is not statistically significance (Supplementary Figure S1A). Furthermore, the serum levels of liver enzymes: ALT and AST as well as other general markers of toxicity such as Creatinine and Albumin were comparable among all treatment groups (Supplementary Figure S1B) indicating its relative tolerability of the regimen. When the same treatment regimens were administered to mice presenting with spontaneous liver tumors, combinatorial treatment again resulted in enhanced control of tumor growth (Supplementary figure S2A) and increased tumor cell death (Supplementary figure S2B).

### Combinatorial treatment with poly-ICLC and Sorafenib reduces tumor cell viability *in vitro*

**Figure 3:**
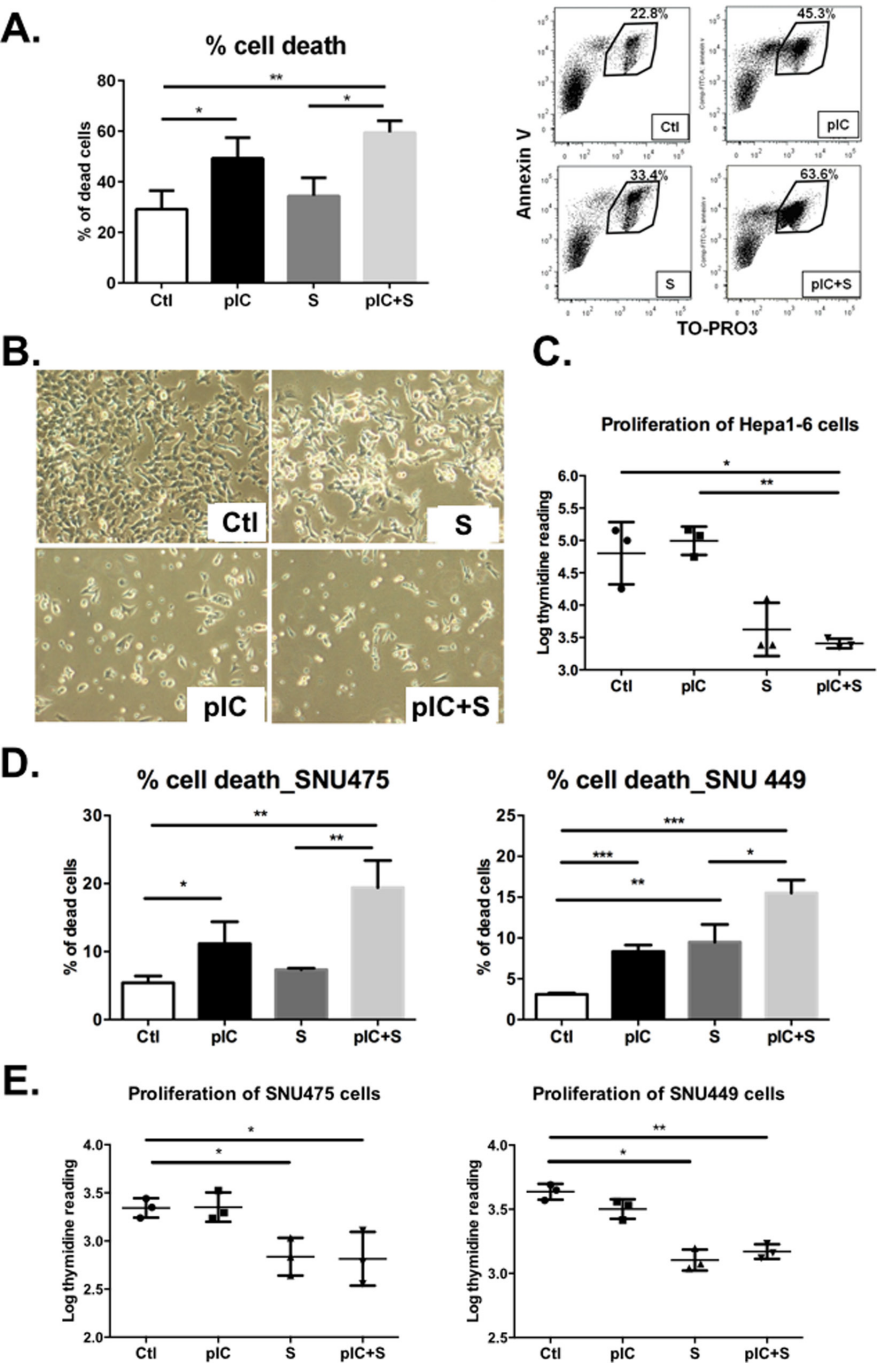
Co-treatment with poly-ICLC and Sorafenib impairs tumor cell survival and proliferation Hepa 1-6 cells were either treated with vehicle (Ctl), poly-ICLC (pIC), Sorafenib (S) or a combination of both (pIC+S) at 37°C. **A.** Enhanced cell death was observed as an increased percentage of Annexin-V^+^Topro-3^+^ cells after 48 h treatment with pIC+S: 59.5 ± 4.6% vs. Ctl: 29.1 ± 7.3%, *p* = 0.004, and vs. S: 34.4 ± 7.1%, *p* = 0.01. Right, representative dot plots showing increased Annexin-V^+^Topro-3^+^ population among Hepa 1-6 cells treated with pIC+S. **B.** Representative light microscopy images of Hepa 1-6 cells in culture showing enhanced cell death after pIC+S treatment for 48 h (100× magnification). **C.** Inhibition of Hepa 1-6 cell proliferation as measured by thymidine incorporation (Log) at 24 h after treatment with pIC+S: 3.4 ± 0.1 vs. Ctl: 4.8 ± 0.5, *p* = 0.03 and vs. pIC: 4.9 ± 0.2, *p* = 0.005. **D.** Enhanced cell death and **E.** inhibition of cell proliferation were observed in SNU449 and SNU475 cells after treatment with pIC+S. For all graphs, mean and SD were shown. *p < 0.05; **p < 0.01, paired Student's *t*-test, *n* = 3 independent experiments.

The increase in apoptotic cells detected in histology sections of tumors with combinatorial treatment (Figure 2E) suggested that this treatment regimen might have direct effects on the tumor cell viability. To evaluate this possibility, we applied combinatorial treatment directly to tumor cells for 48 h *in vitro* and then measured the proportions of dead cells (Annexin-V and Topro-3-positive cells) by flow cytometry. Enhanced cell death was observed after co-treatment with poly-ICLC and Sorafenib (Figure 3A). Microscopy images of the cells in culture also confirmed an increase in tumor cell death after combinatorial treatment (Figure 3B). Drug-induced cell death was detectable as soon as 24 h after treatment (data not shown), but the pro-apoptotic effects of co-treatment were more pronounced after 48 h. The combinatorial treatment approach also inhibited the proliferation of Hepa 1-6 cells effectively (Figure 3C). The inhibition of tumor cell proliferation at 24 h preceded the substantial increase in cell death observed at 48 h. The increased cell death and inhibition of proliferation is consistent with the *in vivo* observation of reduced tumor growth and final tumor mass in mice treated with the combinatorial therapy. Similar effects on tumor cell death and proliferation were observed in two other human HCC cell lines: SNU449 and SNU475 (Figure 3D and 3E). Intriguingly, the observed drug effect on tumor cell death was predominantly induced by poly-ICLC (except for SNU449 which is sensitive to Sorafenib induced cell death), whereas tumor cell proliferation appeared to be inhibited mainly by Sorafenib, observed by these individual effects in tumor cells treated with the corresponding monotherapies. We also tested the effect of the combinatorial treatment on one normal liver cell line, THLE-2 and the apoptotic effect was not observed (data not shown).

### Combinatorial treatment with poly-ICLC and Sorafenib inhibits AKT and MEK/ERK pathways *in vitro*

**Figure 4:**
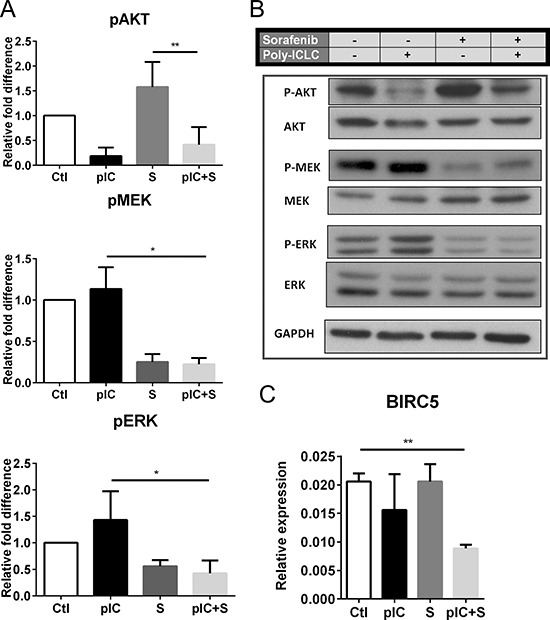
Co-treatment with Sorafenib and poly-ICLC impairs phosphorylation of AKT, MEK and ERK in Hepa 1-6 cells Hepa 1-6 cells were treated with vehicle (Ctl), poly-ICLC (pIC), Sorafenib (S) or combination of both (pIC+S) at 37°C. **A.** Inhibition of pAKT, pMEK and pERK in cells 24 h post-treatment was revealed by densitometry measurement of phospho-protein expression relative to total protein levels. Graphs show the relative fold-change of the various treatments relative to Ctl. **B.** Representative Western blot images showing expression of phosphorylated proteins with respect to total protein levels and expression of the housekeeping protein GAPDH. **C.** Reduced relative gene expression of BIRC5 with respect to the housekeeping gene GAPDH, as assessed by quantitative-PCR in cells treated with pIC+S for 8 h. For all graphs, mean and SD are shown. **p < 0.01, paired Student's *t*-test, *n* = 3 independent experiments.

The signaling proteins AKT, MEK and ERK mediate critical tumor cell apoptosis and proliferation pathways [20–23], so we next investigated whether the inhibitory effects of poly-ICLC/Sorafenib combinatorial therapy could be associated with modulation of these key pathways. To do this, Hepa 1-6 cells were treated with either monotherapies or combinatorial therapy for 24 h and we observed that phosphorylations of AKT (Ser473), MEK1/2 (Ser217/221) and ERK1/2 (Thr202/Tyr204) were inhibited by co-treatment (Figure 4A and 4B). Poly-ICLC appeared to be primarily responsible for the reduced phosphorylation of AKT, whereas Sorafenib for the decreased phosphorylation of MEK1/2 and ERK1/2 (Figure 4A and 4B). These data were in-line with our previous observations that drug effects on tumor cell death could be attributed to poly-ICLC, while the effect on proliferation was mainly induced by Sorafenib (Figure 3). Importantly, our data also indicated that combining Sorafenib and poly-ICLC did not interfere with the action of the individual drugs and instead inhibited all three kinase pathways in parallel to exert potent effects on tumor cell growth and survival. However modulations on the signaling pathways were inconclusive in both SNU449 and SNU475 cell lines (data not shown). Consistent with these data, combinatorial treatment also reduced tumor cell expression of the Survivin gene (BIRC5) (Figure 4C), which has previously been implicated in tumor cell survival [24, 25].

### Combining poly-ICLC and Sorafenib enhances NK cells and T cells activation *in vivo*

**Figure 5:**
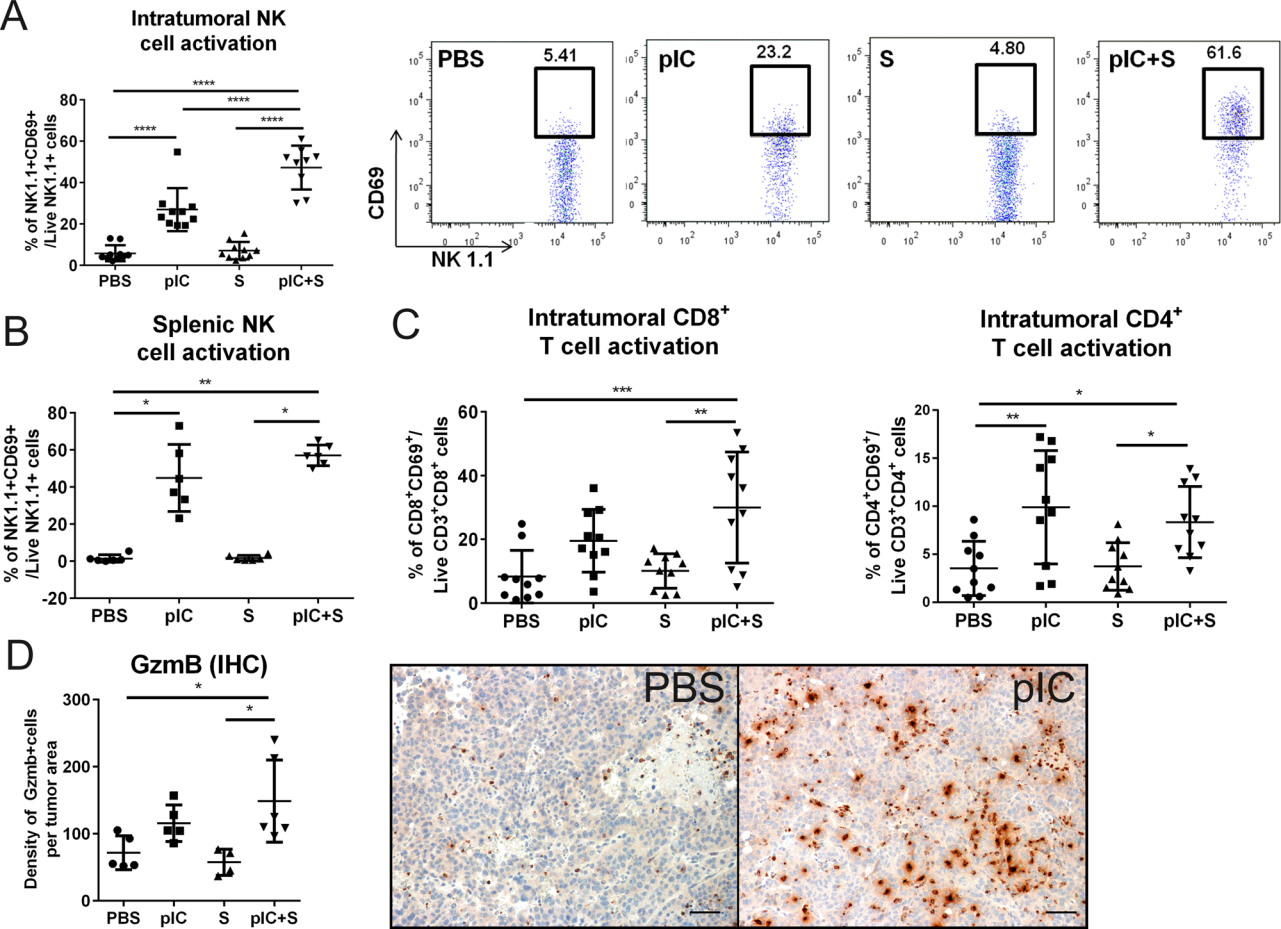
Enhanced activation of host NK cells and T cells in co-treated tumor-bearing mice C57BL/6 mice were transplanted with Hepa 1-6 cells and treated as indicated in Figure 2C. Increased percentages of activated CD69^+^NK1.1^+^ NK cells were detected in pIC+S-treated mice in **A.** tumors (*n* = 8–10); pIC+S: 47.3 ± 10.6% vs. PBS: 5.9 ± 3.8%, *p* < 0.0001; vs. pIC: 27.0 ± 10.4%, *p* < 0.0001 and vs. S: 7.1 ± 4.1%, *p* < 0.0001; and in **B.** spleens (*n* = 6); pIC+S: 57.1 ± 5.6% vs. PBS: 1.4 ± 2.0%, *p* = 0.002; and vs. S: 1.8 ± 1.4%, *p* = 0.017. A, right, Representative dot plots showing synergistic increases in the percentage of intratumoral CD69^+^ activated NK cells in mice treated with pIC+S. **C.** Increased percentage of CD8^+^CD69^+^: pIC+S: 30.0 ± 17.4% vs. PBS: 8.4 ± 8.2%, *p* = 0.0006 and vs. S: 10.1 ± 5.4%, *p* = 0.0017; and CD4^+^CD69^+^: pIC+S: 8.3 ± 3.7% vs. PBS: 3.5 ± 2.8%, *p* = 0.04 and vs. S: 3.7 ± 2.5%, *p* = 0.04 (*n* = 8–10) **D.** Left, Increased density of Granzyme-B^+^ (GzmB) cells per tumor field in mice treated with pIC+S: 149 ± 61 vs. PBS: 72 ± 25, *p* = 0.04 and vs. S: 58 ± 19, *p* = 0.01 (*n* = 4–6). Right, Representative immunohistochemistry images of Granzyme-B staining (brown) (200× magnification, scale bar = 50 μm). For all graphs, mean and SD are shown. Points represent individual tumors. *p < 0.05; **p < 0.01, ***p < 0.001, ****p < 0.0001. A&C. One-way ANOVA test with post Tukey's multiple comparisons test. B&D. Kruskal-Wallis test with post Dunn's multiple comparisons test.

We have previously reported that TLR3 agonist treatment activates intratumoral NK cells and T cells in mice [11], therefore we next assessed whether these same effects could also be induced by combinatorial therapy in the transplant model of established liver tumors (as described in Figure 2C). Tumor-infiltrating leukocytes (TIL) were isolated from the tumors on d21 after treatment, and we observed increased proportions of activated NK cells that express early activation marker CD69 in co-treated animals, (Figure 5A). Although poly-ICLC monotherapy alone was sufficient to activate NK cells, co-treatment with Sorafenib augmented this effect in a synergistic manner. Indeed, NK cell activation by combinatorial therapy was also observed in the spleen, indicating that co-treatment induced systemic activation of host immune responses, albeit comparable in magnitude to poly-ICLC monotherapy (Figure 5B). Expression of CD69 among CD8^+^ and CD4^+^ T cells within the tumors was comparably increased by poly-ICLC monotherapy and combinatorial therapy (Figure 5C). Of note, TIL density was largely unchanged by the different treatment regimens, although we did observe a significant reduction in CD8^+^ T-cell numbers after poly-ICLC monotherapy (Supplementary figure S3). Consistently, we detected increased expression of Granzyme-B (GzmB), an activation molecule expressed by both cytotoxic T cells and NK cells [26], in the microenvironment of tumors from co-treated mice (Figure 5D). Increased CD69 expression on both CD8^+^ and CD4^+^ T cells was also detected in the spleen (Supplementary Figure S4A). Indeed, in mice that received combinatorial therapy or poly-ICLC monotherapy, T cell activation in the tumor-draining inguinal and axillary lymph nodes was increased relative to the non-draining submandibular and parotid lymph nodes (Supplementary figure S4B), suggesting the induction of tumor-specific T cell responses. Consistently, activation of NK cells and CD8^+^ T cells was observed in the tumors as well as the spleens of spontaneous liver tumor models treated with combinatorial therapy (Supplementary figure S5A and S5B). The therapy-induced activation of NK cells and T cells appeared to be largely induced by poly-ICLC, with little or no contribution from Sorafenib, which is thought to have little immunostimulatory potential.

### Combinatorial treatment with poly-ICLC and Sorafenib restores the tumor immunogenicity *in vivo*

**Figure 6:**
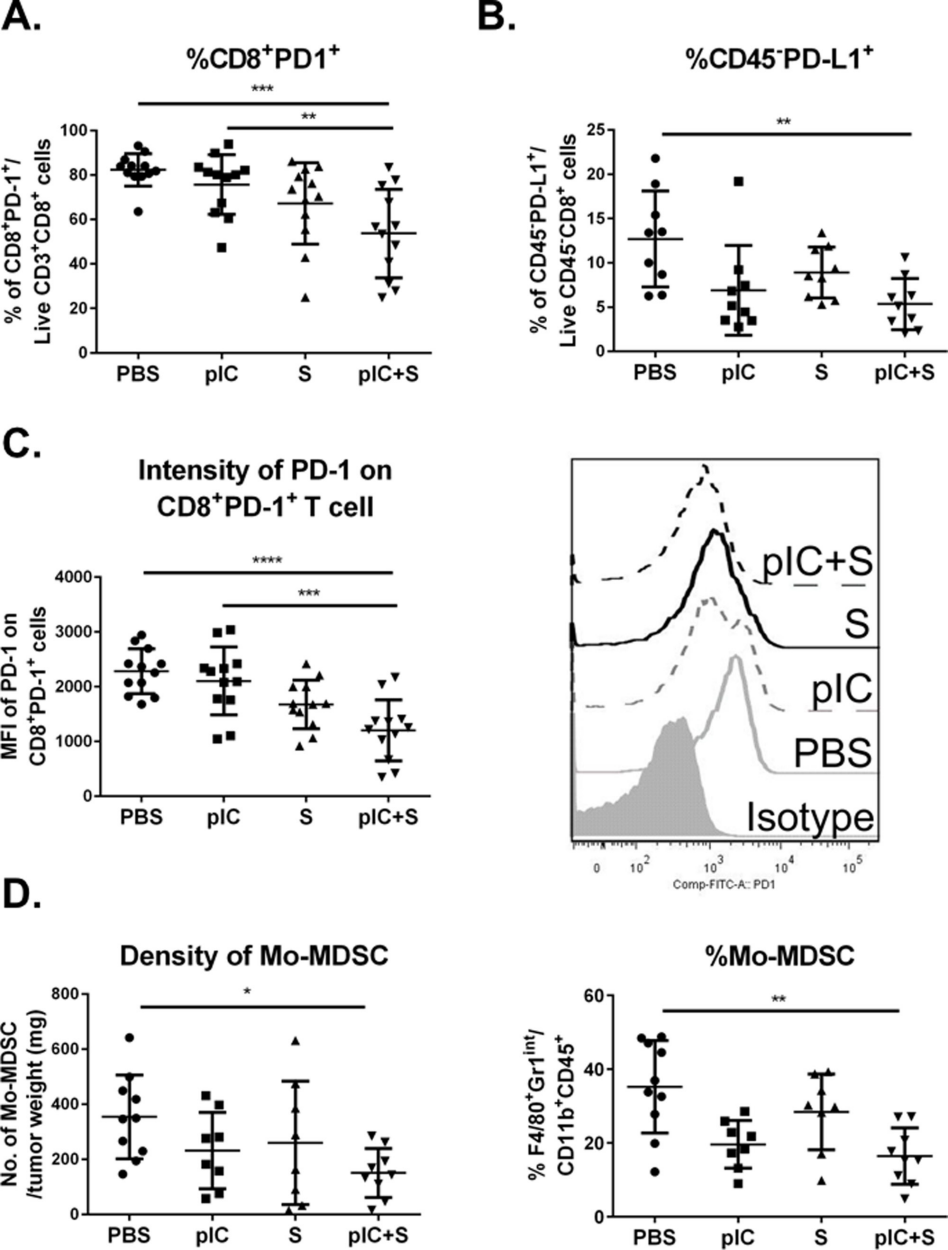
Tumor immunogenicity is enhanced by combinatorial treatment with poly-ICLC and Sorafenib C57BL/6 mice were transplanted with Hepa 1-6 cells and treated as indicated in Figure 2C. Reduced percentage of **A.** PD-1^+^CD8^+^ T cells in mice treated with pIC+S: 53.7 ± 19.9% vs. PBS: 82.3 ± 7.3%, *p* = 0.0003, and vs. pIC: 75.7 ± 13.4%, *p* = 0.0062 and **B.** PD-L1^+^CD45^−^ tumor cells in mice treated with pIC+S: 5.4 ± 2.9% vs. PBS: 12.7 ± 5.4%, *p* = 0.005. **C.** Left, Graph shows reduced median fluorescence intensity (MFI) levels of PD-1 expression on PD-1^+^CD8^+^ T cells isolated from tumors in mice treated with pIC+S: 1202 ± 557 vs. PBS: 2284 ± 411, *p* < 0.0001 and vs. pIC: 2105 ± 621, *p* = 0.0005. Right, Representative histograms showing the intensities of PD-1 staining on CD8^+^ T cells isolated from tumors in mice treated with the various drugs. **D.** Left, Reduced density of Mo-MDSC suppressor cells, indicated as CD45^+^CD11b^+^F4/80^+^Gr1^int^ cells per tumor weight (mg), in tumors from mice treated with pIC+S: 16.5 ± 7.6% vs. PBS: 35.2 ± 12.6%, *p* = 0.001. Right, Reduced percentage of Mo-MDSC among the total CD11b^+^ myeloid population in tumors from mice treated with pIC+S. For all graphs, mean and SD are shown (*n* = 8–10 per treatment group). *p < 0.05; **p < 0.01, ***p < 0.001, ****p < 0.0001, One-way ANOVA test with post Tukey's multiple comparisons test.

Sorafenib has previously been reported to reduce the proportion of CD8^+^PD-1^+^ ‘exhausted’ T cells in the tumor microenvironment [8], therefore we next explored whether combinatorial therapy could potentially enhance this beneficial drug effect. Mice transplanted with Hepa 1-6 cells received the therapeutic regimen described in Figure 2C. We observed a significant reduction in the percentage of CD8^+^ T cells that expressed PD-1 in mice exposed to combinatorial treatment as compared to pIC or PBS control (Figure 6A). Correspondingly, we also observed that co-treatment induced a decrease in the percentage of CD45^−^ tumor cells that expressed the ligand for PD-1, PD-L1 (Figure 6B). The median fluorescence intensity (MFI) of PD-1 expression on these CD8^+^ T cells was also reduced (Figure 6C). These data indicated that combinatorial therapy disrupted the PD-1/PD-L1 immunosuppressive pathway that would otherwise be expected to impair host T cell responses in the tumor microenvironment.

**Figure 7:**
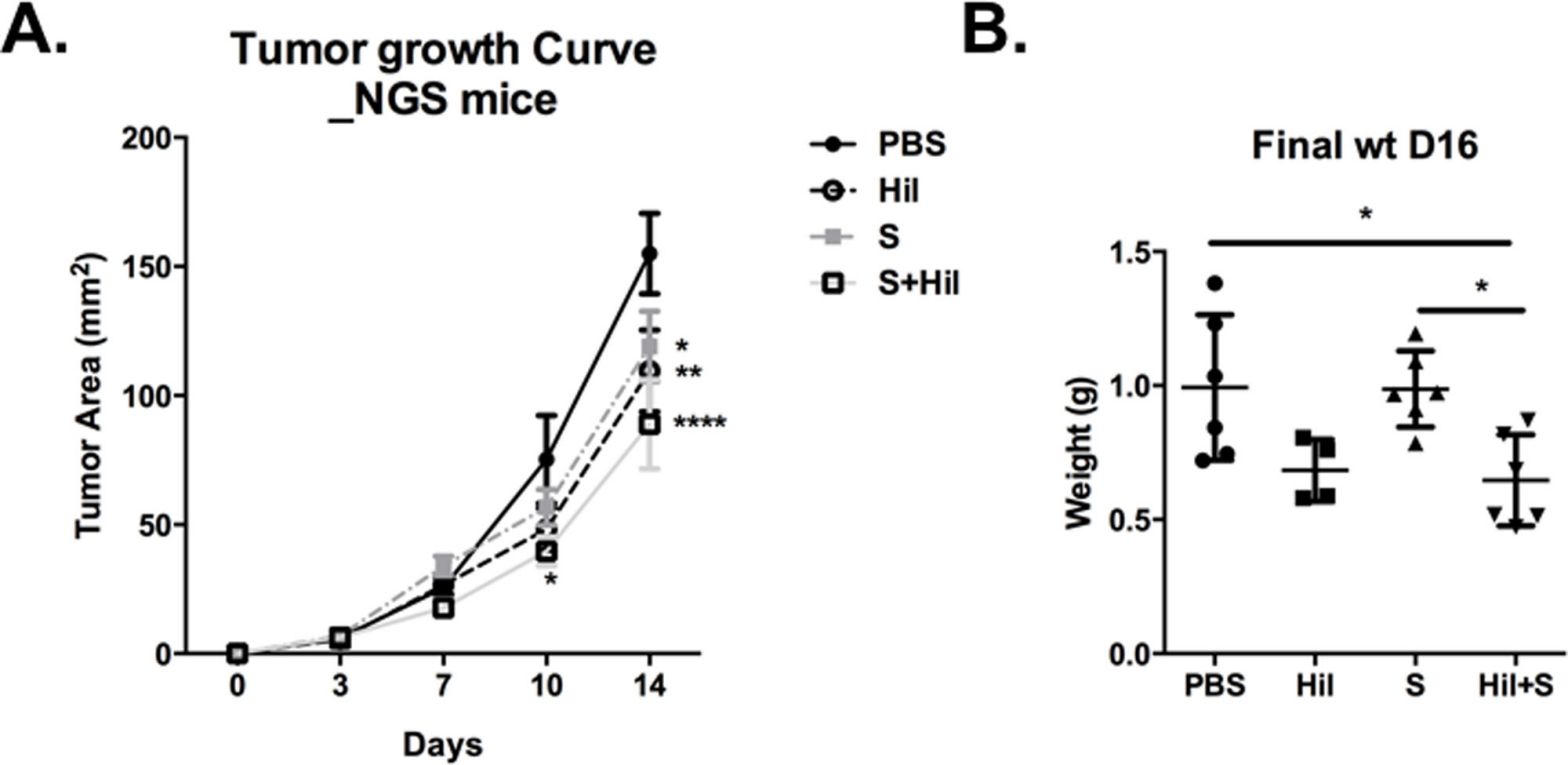
Combination of poly-ICLC and Sorafenib enhanced tumor control in NSG mice NSG mice were transplanted with Hepa 1-6 cells and PBS or poly-ICLC (pIC) were treated as indicated in Figure 2A. **A.** Slowed tumor growth on d10, pIC+S: 39.7 ± 13.8 mm^2^ vs. PBS: 75.3 ± 34.1 mm^2^, *p* = 0.02; on d14 pIC+S: 88.8 ± 42.6 mm^2^ vs. PBS: 155.0 ± 31.1 mm^2^, *p* < 0.0001; Hil: 109.5 ± 31.8 mm^2^ vs PBS, *p* = 0.006; S: 119.0 ± 33.3 mm^2^ vs PBS, *p* = 0.02 and **B.** Decreased final tumor weights (g) as harvested on day 16 in pIC+S-treated mice: 0.65 ± 0.17g vs. PBS: 0.99 ± 0.27, *p* = 0.01; vs. pIC: 0.86 ± 0.08, *p* = ns and vs. S: 0.99 ± 0.014, *p* = 0.01. For all graphs, mean and SD are shown. *p < 0.05; **p < 0.01, ***p < 0.001, ****p < 0.0001. A. Two-way ANOVA with Tukey's multiple comparisons test. B. One-way ANOVA test with post Tukey's multiple comparisons test. Points represent individual tumors.

In addition, we observed that combinatorial treatment reduced the density and percentage of tumor-infiltrating monocytic-myeloid-derived suppressor cells (Mo-MDSC), defined as CD45^+^CD11b^+^F4/80^+^Gr1^int^ cells based on previous reports [27, 28] (Figure 6D). Finally, the combinatorial treatment also enhanced the percentage of activated and migratory DC (CD11c^hi^MHC-II^+^) and macrophages (CD11b^+^F4/80^+^Gr1^−^) as assessed by expression of the activation marker CD80 and migratory marker CCR7 (Supplementary figure S6). To further demonstrate the critical role of immune system in controlling tumor progression in the combinatorial treatment, we repeated the 16-Days treatment regimen on NOD/SCID gamma (NSG) mice transplanted with Hepa 1-6 cells. NSG mice lack mature T cells, B cells, and NK cells [29] as well as reduced dendritic cell function, and defective macrophage activity [30]. We observed better tumor control with combinatorial treatment in these mice (Figure 7A and 7B) but to a lesser extent compared to that in the WT mice (in pIC+S treatment group: the final tumor weight at the end of 16 days treatment is 4 fold smaller in the WT vs NSG teated mice, Figure 7B versus Figure 2B). This shows that this treatment can act independently of immune system via direct killing (which was shown in the *in vitro* data above).

Taken together, these data indicate that combinatorial treatment activates NK-, T- and myeloid-cells while simultaneously disrupting the inhibitory PD-1/PD-L1 pathway and reducing the accumulation of suppressive Mo-MDSC within the tumor microenvironment. The ability of combinatorial therapy to activate host immune responses is likely to contribute to the ability of this novel co-treatment regimen to restrict tumor growth *in vitro* and *in vivo*.

## DISCUSSION

The multi-kinase inhibitor Sorafenib is currently the only FDA-approved drug available for the treatment of advanced HCC [31], and here we demonstrate that the poor clinical efficacy of Sorafenib can be significantly enhanced by simultaneous administration of the TLR3 agonist poly-ICLC. These data are consistent with our previous report that TLR3 agonists are promising candidates for immunotherapy in HCC due to their ability to directly induce tumor cell death and activate host immune responses in the tumor microenvironment [11]. In the current report, we demonstrate that impairment of tumor growth/burden can be significantly enhanced by combining poly-ICLC with Sorafenib as the potent effects on cancer cells and host immune responses cannot be achieved by either drug alone. Our results are also consistent with a recent report showing that combining Sorafenib with a synthetic dsRNA, which could also acts on TLR3, suppresses HCC *in vitro* and *in vivo* [32]. Our data further reveal the mechanisms by which poly-ICLC/Sorafenib co-treatment reduces HCC tumor growth which include direct induction of tumor cell death, inhibition of tumor cell proliferation, and activation of host T cells, NK cells, macrophages and dendritic cells. In parallel, poly-ICLC/Sorafenib co-treatment suppressed the inhibitory PD-1/PD-L1 signaling pathway and restored tumor cell immunogenicity in the local microenvironment.

Indeed combinatorial approach to cancer treatment has proven to be clinically more effective than monotherapy. For example, administration of the TLR3 agonist polyinosinic-polycytidylic acid (poly-IC) together with an antibody against programmed death-ligand 1 (PD-L1) leads to significant improvement in tumor control in mouse models of several different cancers [33]. Likewise, tumor control by Sorafenib treatment can be significantly enhanced by co-administration of the mTOR inhibitor rapamycin [17] or simultaneous use of TACE (transcatheter arterial chemombolization) [16]. In our current study, we observed that combinatorial treatment inhibited tumor cell proliferation via Sorafenib and simultaneously induced tumor cell death via poly-ICLC through their respective effects on the MEK/ERK and AKT pathways. These data were in-line with previous reports that TLR3 ligands can induce apoptosis of human prostate cancer cells via dephosphorylation of AKT [34], and that Sorafenib can impair cell proliferation by inhibiting the MEK/ERK pathway [35]. Although inhibition of AKT, MEK and ERK was not synergistic, the additive effects of combining Sorafenib and poly-ICLC potently restricted tumor cell survival and proliferation. Indeed, previous reports on cancer trials including that in HCC demonstrated that dual inhibition of the PI3K/AKT/mTOR and RAS/MEK/ERK pathways resulted in superior therapeutic responses compared with inhibition of the individual pathways [36]. Even such combinatorial treatment have been reported to confer greater risk of adverse effects, most of these are limited to mild symptoms including rash and hyperglycemia that are usually well tolerated and easily managed [36]. In the current study, we observed marginal weight loss of unknown etiology in the mice that were treated with poly-ICLC alone or combination therapy. However, these mice remained healthy throughout, with no obvious signs of drug toxicity, and were able to regain weight quickly and achieved normal body mass by the end of the therapy. Furthermore, the serum analysis from the mice showed no obvious indications of toxicity.

Enhanced activation of NK cells and T cells was observed after poly-ICLC monotherapy and co-treatment, showing that TLR3 ligation alone was likely to be responsible for these effects; but there was also a synergistic enhancement of NK cell activation with co-treatment when compared with poly-ICLC single treatment. Here, we speculate that enhanced tumor cell death in the presence of both drugs releases more free RNA, which in-turn enhances TLR3 triggering and increases immune activation. Similarly, activation of myeloid cells was only observed in co-treated mice, likely as a result of enhanced tumor killing leading to increased release of ‘danger signals’ that activate these cells [37]. It is therefore possible that enhanced tumor cell death in the presence of co-treatment, either by direct killing or via immune activation, establishes a positive feedback loop that further enhances beneficial anti-tumor immune responses which could potentially improve patient outcomes in HCC.

Despite reported immuno-suppressive effects [6, 7], Sorafenib has also been shown to reduce the proportion of PD-1-expressing CD8^+^ T cells within the tumor microenvironment in orthotopic HCC mice [8]. This result is consistent with the current study in which we observed a reduced percentage of PD-1-expressing CD8^+^ T cells in tumor-bearing mice after treatment with Sorafenib alone. In co-treated animals however the percentage and level of PD-1 expression by CD8^+^ T cells was further reduced, and also accompanied by a corresponding decrease in tumor cell expression of PD-L1. Since the PD-1/PD-L1 pathway exerts immunosuppressive effects within the tumor microenvironment and promotes malignant progression [38], disruption of this pathway by combinatorial treatment is likely to induce beneficial anti-tumor effects. Another recent report using triple drugs strategy with anti-PD-1 antibody and CXCR4 inhibitor combined with Sorafenib demonstrated the importance of anti-PD-1 in the enhanced immune activation of tumor microenvironment leading to superior control in tumor growth [29]. Besides the suppression of PD-1/PD-L1 pathway, our current study further demonstrated that tumor-infiltration by Mo-MDSC was reduced in the poly-ICLC and Sorafenib co-treated mice. Taken together, these observations highlights the potential benefits of using combinatiorial treatment to restore the immunogenicity of tumors so that the host immune system can mount effective anti-cancer responses.

Our current findings indicate that combining poly-ICLC with Sorafenib therapy not only enhances direct tumor killing and impairs tumor cell proliferation, but also enhances host immune responses that may contribute to the efficacy of this novel co-treatment regimen.

## MATERIALS AND METHODS

### Mice, cell lines and reagents

Male C57BL/6 (B6) and NOD/SCID gamma (NSG) mice were obtained from the Biological Resource Centre, Agency for Science, Technology and Research (A*STAR), Singapore. Animal care and all experimental procedures were approved by the Institutional Animal Care and Use Committee. The murine hepatoma cell line Hepa 1-6 and two human HCC cell lines SNU449 and SNU475 were obtained from ATCC. TLR3 agonist lysine-stabilized poly-IC (poly-ICLC or Hiltonol™) was provided by Andres Salazar (Oncovir, Inc.). Sorafenib or Nexavar^®^ 200 mg film-coated tablets were purchased from Bayer AG and was used at 60 μg/g of Harlan diet. This dose was based on assessment of food consumption by age-matched mice and is equivalent to Sorafenib administration at 10 mg/kg of body weight per day [35]. A trial was performed to show equivalent effect of Sorafenib given either with oral gavage or diet (data not shown). For *in vitro* experiments, Sorafenib tablets were dissolved in DMSO and further diluted in culture media to obtain the final concentrations indicated below.

### Cell death and proliferation assay

For cell death assays, 3 × 10^5^ cells were cultured with 50 μg/ml poly-ICLC and 15 μM Sorafenib either alone or in combination for 48 h at 37°C. The cells were then harvested by addition of 0.5 mM EDTA in PBS to each well and apoptotic HCC cells were identified by staining with Annexin V (BD Pharmingen) and Topro-3 (Invitrogen) according to manufacturer's protocol, followed by analysis using a BD FACSCanto flow cytometer (BD Biosciences,) and FlowJo software (version 7.6.5, Tree Star Inc.).

For cell proliferation assays, 5 × 10^4^ cells were cultured with the above-mentioned treatments for 24 h at 37°C. A total of 2.5 μCi/ml ^3^H-thymidine (Perkin Elmer) was added to the cell culture for the final 18 h of culture and cell proliferation was determined using a FilterMate Harvester (Perkin Elmer).

### Western blot analysis and quantitative PCR

6 × 10^5^ Hepa 1-6 cells were incubated with 50 μg/ml poly-ICLC and 10 μM Sorafenib either alone or in combination for 24 h at 37°C. The cells were then collected for protein extraction using RIPA buffer (Millipore) containing protease inhibitors (Roche) and phosphatase inhibitors (Santa Cruz Biotechnology). Protein extracts were separated by SDS-PAGE and transferred onto PVDF membranes before being probed with antibodies indicated in Supplementary Table S1. Proteins of interest were detected using HRP-conjugated secondary antibodies together with Pierce ECL Western blotting substrate (Thermo Scientific) according to the provided protocol. Relative protein expression levels were determined by densitometry using ImageJ software (NIH) and the relative fold differences were calculated with respect to the untreated control.

For quantitative PCR (qPCR) assay, 3 × 10^5^ Hepa 1-6 cells were cultured with the above-mentioned treatments for 8 h at 37°C. The cells were then collected for qPCR analysis of survivin/BIRC5 RNA expression. BIRC5 primers: FW'ATCGCCACCTTCAAGAACTG; RV'ATCAGGCTCGTTCTCGGTAG. Relative RNA expression of BIRC5 was normalized to the housekeeping gene GAPDH: FW' GCCGGTGCTGAGTATGTCGT; RV' GGAGATGATGACCCGTTTGG.

### Tumor models and treatment protocols

For the transplanted model, 5–6 week old B6 Mice were injected subcutaneously with 5 × 10^6^ Hepa 1-6 cells on both sides of the shaved rear flank. 20 μl of poly-ICLC (40 μg/dose) or endotoxin-free sterile PBS (Hyclone) were delivered intramuscularly (IM) as established by previous studies [39] starting 1d after cell transfer, followed by further injections on d3, d7, d10, and d14. Alternatively, treatment was delayed until 6d after cell transfer to allow tumor growth to an average size of 10 mm^2^, followed by poly-ICLC/PBS injections on d8, d10, d13, d16, and d20. As indicated, some animals also received dietary supplementation with Sorafenib throughout the duration of the poly-ICLC/PBS therapy. Mouse food consumption was monitored and corresponded to a dose of 8.5–11.5 μg Sorafenib per gram of body weight per day. Calipers were used to measure tumor area (mm^2^) on the same days of drugs injections, and final tumor weight was recorded upon harvesting on d16 (immediate treatment) or d21 (delayed treatment).

For the spontaneous tumor model, we used the *Sleeping Beauty* transposon system to induce liver tumorigenesis as described previously [40]. Briefly, 7–9 week old B6 mice were injected with a plasmid cocktail in a volume corresponding to 10% of their body weight (e.g. 2 ml for a 20 g mouse) using 27-gauge needles inserted into the lateral tail vein and discharged over a period of 8–10 s. Plasmid DNAs were purified using EndoFree Plasmid Maxi kits (Qiagen) and diluted in each 2.5 ml of lactated Ringer's solution with transposase (15 μg pGK-SleepingBeauty13) and transposon (30 μg pT2-Caggs-NRasV12 and 15 μg pT2-shRNAp53). 10–12 weeks after tumor induction, poly-ICLC/PBS was administered IM (same dose as above) on alternate days for the first 3 doses, and then twice weekly thereafter for a further 3 weeks (some animals received Sorafenib diet throughout the period). Tumor volume (mm^3^) was monitored using a Bruker Clinscan 7T magnetic resonance imaging (MRI) scanner. After the localizer, a T1-weighted magnetization-prepared rapid gradient echo (MPRAGE) and a T2-weighted Turbo Spin Echo (TSE) were performed. The T1- and T2-weighted scans were used for tumor identification and tumor volume were measured from the images acquired with T1-weighted MPRAGE. Liver tumors and non-tumorous liver were harvested and weighed on the day after the final treatment.

### Flow cytometry and immunohistochemistry

Total leukocytes were isolated from the tumors, spleens, draining and non-draining lymph nodes by digesting the tissues with 1 mg/ml Collagenase A and 0.1 mg/ml DNase I (Roche). The immune cells were then labelled with the antibodies listed in Supplementary Table S1 and analyzed using a BD LSRII flow cytometer equipped with five lasers (BD Biosciences,), together with FlowJo software (version 7.6.5, Tree Star Inc.).

Immunohistochemistry was performed as previously reported [10]. Primary antibodies used are listed in Supplementary Table S1. The Dako-Cytomation EnVision+HRP System was used to reveal antibody staining. Terminal deoxynucleotidyl transferase dUTP nick end labeling (TUNEL) assay was performed according to the manufacturer's (Millipore) protocol. Quantification of stained cells was performed using 5–10 random fields at X100 magnification in ImagePro software. The data presented are the mean number of stained cells per field per sample.

### Statistical analysis

Data were evaluated using paired/unpaired student's *t*-tests, Mann-Whitney tests, One-way/two-way ANOVA, and Kruskal-Wallis tests with post-hoc multiple comparisons as indicated in the text. *P*-values < 0.05 were considered significant. All statistical tests were two-sided. All analyses and graphs were conducted/created using GraphPad Prism 6.03 (GraphPad Software).

## SUPPLEMENTARY TABLE AND FIGURES



## Abbreviations

HCC: Hepatocellular carcinoma
TLR-3: Toll-like receptor-3
poly-ICLC: lysine-stabilized polyinosinic-polycytidylic-acid
NK: natural killer cells
DC: dendritic cells
TIL: Tumor-infiltrating leukocytes
Mo-MDSC: monocytic-myeloid-derived suppressor cells

## References

[1] Ferlay J, Soerjomataram I, Dikshit R, Eser S, Mathers C, Rebelo M, Parkin DM, Forman D, Bray F (2014). Cancer incidence and mortality worldwide: Sources, methods and major patterns in GLOBOCAN 2012. Int J Cancer.

[2] Llovet JM, Ricci S, Mazzaferro V, Hilgard P, Gane E, Blanc JF, de Oliveira AC, Santoro A, Raoul JL, Forner A, Schwartz M, Porta C, Zeuzem S, Bolondi L, Greten TF, Galle PR (2008). Sorafenib in advanced hepatocellular carcinoma. N Engl J Med.

[3] Chow AK, Ng L, Lam CS, Wong SK, Wan TM, Cheng NS, Yau TC, Poon RT, Pang RW (2013). The Enhanced metastatic potential of hepatocellular carcinoma (HCC) cells with sorafenib resistance. PLoS One.

[4] van Malenstein H, Dekervel J, Verslype C, Van Cutsem E, Windmolders P, Nevens F, van Pelt J (2013). Long-term exposure to sorafenib of liver cancer cells induces resistance with epithelial-to-mesenchymal transition, increased invasion and risk of rebound growth. Cancer Lett.

[5] Wilhelm SM, Adnane L, Newell P, Villanueva A, Llovet JM, Lynch M (2008). Preclinical overview of sorafenib, a multikinase inhibitor that targets both Raf and VEGF and PDGF receptor tyrosine kinase signaling. Mol Cancer Ther.

[6] Hipp MM, Hilf N, Walter S, Werth D, Brauer KM, Radsak MP, Weinschenk T, Singh-Jasuja H, Brossart P (2008). Sorafenib, but not sunitinib, affects function of dendritic cells and induction of primary immune responses. Blood.

[7] Zhang QB, Sun HC, Zhang KZ, Jia QA, Bu Y, Wang M, Chai ZT, Wang WQ, Kong LQ, Zhu XD, Lu L, Wu WZ, Wang L, Tang ZY (2013). Suppression of natural killer cells by sorafenib contributes to prometastatic effects in hepatocellular carcinoma. PLoS One.

[8] Chen ML, Yan BS, Lu WC, Chen MH, Yu SL, Yang PC, Cheng AL (2014). Sorafenib relieves cell-intrinsic and cell-extrinsic inhibitions of effector T cells in tumor microenvironment to augment antitumor immunity. Int J Cancer.

[9] Chew V, Chen J, Lee D, Loh E, Lee J, Lim KH, Weber A, Slankamenac K, Poon RT, Yang H, Ooi LL, Toh HC, Heikenwalder M, Ng IO, Nardin A, Abastado JP (2012). Chemokine-driven lymphocyte infiltration: an early intratumoural event determining long-term survival in resectable hepatocellular carcinoma. Gut.

[10] Chew V, Tow C, Teo M, Wong HL, Chan J, Gehring A, Loh M, Bolze A, Quek R, Lee VK, Lee KH, Abastado JP, Toh HC, Nardin A (2010). Inflammatory tumour microenvironment is associated with superior survival in hepatocellular carcinoma patients. J Hepatol.

[11] Chew V, Tow C, Huang C, Bard-Chapeau E, Copeland NG, Jenkins NA, Weber A, Lim KH, Toh HC, Heikenwalder M, Ng IO, Nardin A, Abastado JP (2012). Toll-like receptor 3 expressing tumor parenchyma and infiltrating natural killer cells in hepatocellular carcinoma patients. J Natl Cancer Inst.

[12] Salaun B, Coste I, Rissoan MC, Lebecque SJ, Renno T (2006). TLR3 can directly trigger apoptosis in human cancer cells. J Immunol.

[13] Salaun B, Lebecque S, Matikainen S, Rimoldi D, Romero P (2007). Toll-like receptor 3 expressed by melanoma cells as a target for therapy?. Clin Cancer Res.

[14] Morikawa T, Sugiyama A, Kume H, Ota S, Kashima T, Tomita K, Kitamura T, Kodama T, Fukayama M, Aburatani H (2007). Identification of Toll-like receptor 3 as a potential therapeutic target in clear cell renal cell carcinoma. Clin Cancer Res.

[15] Galluzzi L, Vacchelli E, Eggermont A, Fridman WH, Galon J, Sautes-Fridman C, Tartour E, Zitvogel L, Kroemer G (2012). Trial Watch: Experimental Toll-like receptor agonists for cancer therapy. Oncoimmunology.

[16] Erhardt A, Kolligs F, Dollinger M, Schott E, Wege H, Bitzer M, Gog C, Lammert F, Schuchmann M, Walter C, Blondin D, Ohmann C, Haussinger D (2014). TACE plus sorafenib for the treatment of hepatocellular carcinoma: results of the multicenter, phase II SOCRATES trial. Cancer Chemother Pharmacol.

[17] Newell P, Toffanin S, Villanueva A, Chiang DY, Minguez B, Cabellos L, Savic R, Hoshida Y, Lim KH, Melgar-Lesmes P, Yea S, Peix J, Deniz K, Fiel MI, Thung S, Alsinet C (2009). Ras pathway activation in hepatocellular carcinoma and anti-tumoral effect of combined sorafenib and rapamycin *in vivo*. J Hepatol.

[18] Jiang X, Feng K, Zhang Y, Li Z, Zhou F, Dou H, Wang T (2015). Sorafenib and DE05, a novel c-Met inhibitor, synergistically suppress hepatocellular carcinoma. Oncotarget.

[19] Giovannini C, Baglioni M, Baron Toaldo M, Ventrucci C, D'Adamo S, Cipone M, Chieco P, Gramantieri L, Bolondi L (2013). Notch3 inhibition enhances sorafenib cytotoxic efficacy by promoting GSK3b phosphorylation and p21 down-regulation in hepatocellular carcinoma. Oncotarget.

[20] Chambard JC, Lefloch R, Pouyssegur J, Lenormand P (2007). ERK implication in cell cycle regulation. Biochim Biophys Acta.

[21] Engelman JA (2009). Targeting PI3K signalling in cancer: opportunities, challenges and limitations. Nat Rev Cancer.

[22] Roberts PJ, Der CJ (2007). Targeting the Raf-MEK-ERK mitogen-activated protein kinase cascade for the treatment of cancer. Oncogene.

[23] Song G, Ouyang G, Bao S (2005). The activation of Akt/PKB signaling pathway and cell survival. J Cell Mol Med.

[24] Cao L, Li C, Shen S, Yan Y, Ji W, Wang J, Qian H, Jiang X, Li Z, Wu M, Zhang Y, Su C (2013). OCT4 increases BIRC5 and CCND1 expression and promotes cancer progression in hepatocellular carcinoma. BMC Cancer.

[25] Wang C, Zheng X, Shen C, Shi Y (2012). MicroRNA-203 suppresses cell proliferation and migration by targeting BIRC5 and LASP1 in human triple-negative breast cancer cells. J Exp Clin Cancer Res.

[26] Trapani JA, Sutton VR, Granzyme B (2003). pro-apoptotic, antiviral and antitumor functions. Curr Opin Immunol.

[27] Schlecker E, Stojanovic A, Eisen C, Quack C, Falk CS, Umansky V, Cerwenka A (2012). Tumor-infiltrating monocytic myeloid-derived suppressor cells mediate CCR5-dependent recruitment of regulatory T cells favoring tumor growth. J Immunol.

[28] Toh B, Wang X, Keeble J, Sim WJ, Khoo K, Wong WC, Kato M, Prevost-Blondel A, Thiery JP, Abastado JP (2011). Mesenchymal transition and dissemination of cancer cells is driven by myeloid-derived suppressor cells infiltrating the primary tumor. PLoS Biol.

[29] Shultz LD, Lyons BL, Burzenski LM, Gott B, Chen X, Chaleff S, Kotb M, Gillies SD, King M, Mangada J, Greiner DL, Handgretinger R (2005). Human lymphoid and myeloid cell development in NOD/LtSz-scid IL2R gamma null mice engrafted with mobilized human hemopoietic stem cells. J Immunol.

[30] Shultz LD, Schweitzer PA, Christianson SW, Gott B, Schweitzer IB, Tennent B, McKenna S, Mobraaten L, Rajan TV, Greiner DL (1995). Multiple defects in innate and adaptive immunologic function in NOD/LtSz-scid mice. J Immunol.

[31] Kim HY, Park JW, Nam BH, Kim HK, Choi JI, Kim TH, Kim HB, Kim CM (2011). Survival of patients with advanced hepatocellular carcinoma: sorafenib versus other treatments. J Gastroenterol Hepatol.

[32] Xu YY, Chen L, Wang GL, Zhou JM, Zhang YX, Wei YZ, Zhu YY, Qin J (2013). A synthetic dsRNA, as a TLR3 pathwaysynergist, combined with sorafenib suppresses HCC *in vitro* and *in vivo*. BMC Cancer.

[33] Nagato T, Lee YR, Harabuchi Y, Celis E (2014). Combinatorial immunotherapy of polyinosinic-polycytidylic acid and blockade of programmed death-ligand 1 induce effective CD8 T-cell responses against established tumors. Clin Cancer Res.

[34] Harashima N, Inao T, Imamura R, Okano S, Suda T, Harada M (2012). Roles of the PI3K/Akt pathway and autophagy in TLR3 signaling-induced apoptosis and growth arrest of human prostate cancer cells. Cancer Immunol Immunother.

[35] Liu L, Cao Y, Chen C, Zhang X, McNabola A, Wilkie D, Wilhelm S, Lynch M, Carter C (2006). Sorafenib blocks the RAF/MEK/ERK pathway, inhibits tumor angiogenesis, and induces tumor cell apoptosis in hepatocellular carcinoma model PLC/PRF/5. Cancer Res.

[36] Shimizu T, Tolcher AW, Papadopoulos KP, Beeram M, Rasco DW, Smith LS, Gunn S, Smetzer L, Mays TA, Kaiser B, Wick MJ, Alvarez C, Cavazos A, Mangold GL, Patnaik A (2012). The clinical effect of the dual-targeting strategy involving PI3K/AKT/mTOR and RAS/MEK/ERK pathways in patients with advanced cancer. Clin Cancer Res.

[37] Aymeric L, Apetoh L, Ghiringhelli F, Tesniere A, Martins I, Kroemer G, Smyth MJ, Zitvogel L (2010). Tumor cell death and ATP release prime dendritic cells and efficient anticancer immunity. Cancer Res.

[38] Wei S, Shreiner AB, Takeshita N, Chen L, Zou W, Chang AE (2008). Tumor-induced immune suppression of *in vivo* effector T-cell priming is mediated by the B7-H1/PD-1 axis and transforming growth factor beta. Cancer Res.

[39] Salazar AM, Erlich RB, Mark A, Bhardwaj N, Herberman RB (2014). Therapeutic In Situ Autovaccination against Solid Cancers with Intratumoral Poly-ICLC: Case Report, Hypothesis, and Clinical Trial. Cancer Immunol Res.

[40] Bell JB, Podetz-Pedersen KM, Aronovich EL, Belur LR, McIvor RS, Hackett PB (2007). Preferential delivery of the Sleeping Beauty transposon system to livers of mice by hydrodynamic injection. Nat Protoc.

