# Supplementary material for: TLR3 agonist and Sorafenib combinatorial therapy promotes immune activation and controls hepatocellular carcinoma progression
Source: Oncotarget. 2015 Jul 23;6(29):27252–66. doi: 10.18632/oncotarget.4583 (PMC4694987; doi:10.18632/oncotarget.4583)
Supplement: Supplementary file 1 [file oncotarget-06-27252-s001.pdf]

SUPPLEMENTARY TABLE AND FIGURES

Reagents and antibodies

The list of antibodies used in immunohistochemistry (IHC), flow cytometry (FC) and western blotting (WB) was listed in Supplementary Table S1 below. The working dilutions are 1:50 for all antibodies.

Supplementary Table S1: List of antibodies

Antibody	Host	Clone	Company and location	Application
CD279 (PD-1)	Rat	RMP1-30	BD Biosciences, San Jose, CA	FC
CD274 (PDL-1)	Rat	MIH5	BD Biosciences, San Jose, CA	FC
CD80	Hamster	16-10A1	BD Biosciences, San Jose, CA	FC
CD40	Rat	3/23	BD Biosciences, San Jose, CA	FC
CD197 (CCR7)	Rat	4B12	eBiosciences, San Diego, CA	FC
CD69	Hamster	H1.2F3	Biolegend, San Diego, CA	FC
CD45	Rat	30-F11	Invitrogen, Carlsbad, CA	FC
CD3	Rat	RM4-5	eBiosciences, San Diego, CA	FC
CD8	Rat	5H10	Invitrogen, Carlsbad, CA	FC
CD4	Rat	GK1.5	Biolegend, San Diego, CA	FC
NK1.1	Mouse	PK136	BD Biosciences, San Jose, CA	FC
CD11b	Rat	M1/70	eBiosciences, San Diego, CA	FC
CD11c	Hamster	N418	Biolegend, San Diego, CA	FC
Gr1	Rat	RB6-8C5	Biolegend, San Diego, CA	FC
I-A/I-E	Rat	M5/114.15.2	BD Biosciences, San Jose, CA	FC
F4/80	Rat	BM8	eBiosciences, San Diego, CA	FC

(*Continued*)

Antibody	Host	Clone	Company and location	Application
CD3	Rat	KT3	Acris Antibodies GmbH, Herford, Germany.	IHC
Granyzme B	Goat	Polyclonal	R&D Systems, Inc.	IHC
Phospho-AKT (Ser 473)	Rabbit	D9E	Cell Signaling Technology, Danvers, MA	WB
Phospho-p44/42 MAPK (Thr202/ Tyr204)	Rabbit	D13.14.4E	Cell Signaling Technology, Danvers, MA	WB
Phospho-MEK1/2 (Ser217/221)	Rabbit	Polyclonal	Cell Signaling Technology, Danvers, MA	WB
Pan AKT	Mouse	40D4	Cell Signaling Technology, Danvers, MA	WB
p44/42 MAPK	Mouse	L34F12	Cell Signaling Technology, Danvers, MA	WB
MEK 1/2	Mouse	L38C12	Cell Signaling Technology, Danvers, MA	WB
GAPDH	Chicken	Polyclonal	Millipore, Billerica, MA	WB

A.

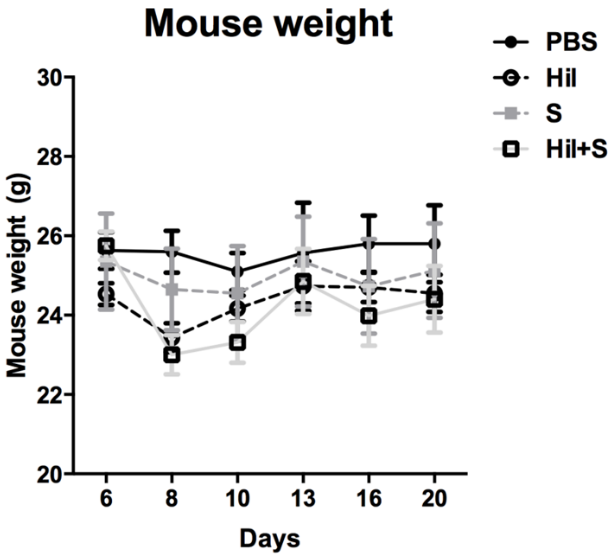

Supplementary Figure S1: Low toxicity shown in mice treated with combinatorial treatment. C57BL/6 mice were transplanted with Hepa 1-6 cells in each flank and treated with either PBS, poly-ICLC (pIC), Sorafenib (S) or combination of both (pIC+S) as indicated in Figure 2C. **A.** An initial loss of body weight was noted in mice that were treated with either pIC or pIC+S, but p = ns (not significant). (*Continued*)

B.

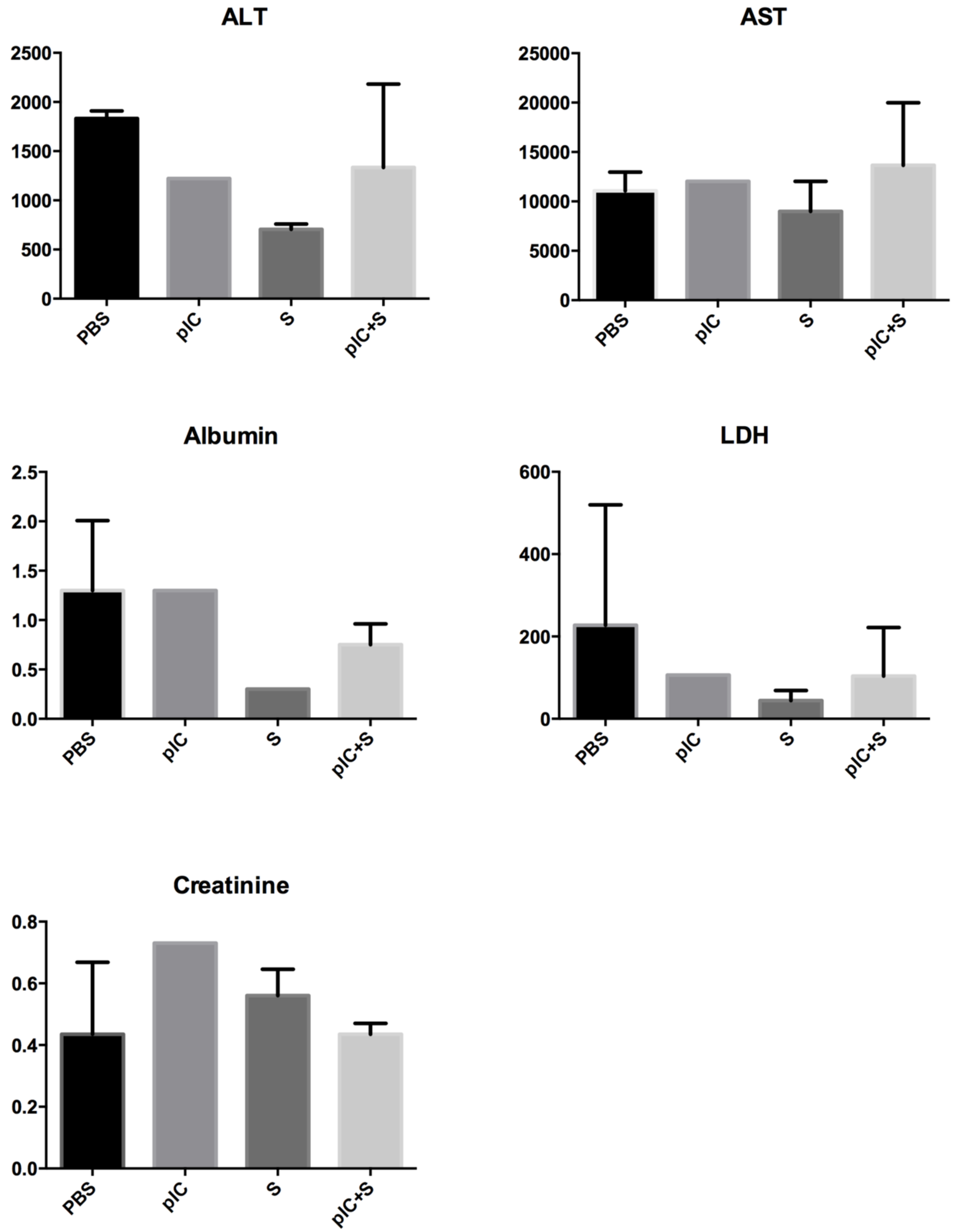

Supplementary Figure S1: (*Continued*) Low toxicity shown in mice treated with combinatorial treatment. B. Serum level of ALT, AST, Albumin, LDH and Creatinine were comparable among all treatment groups One-way ANOVA test with post Tukey's multiple comparisons test.

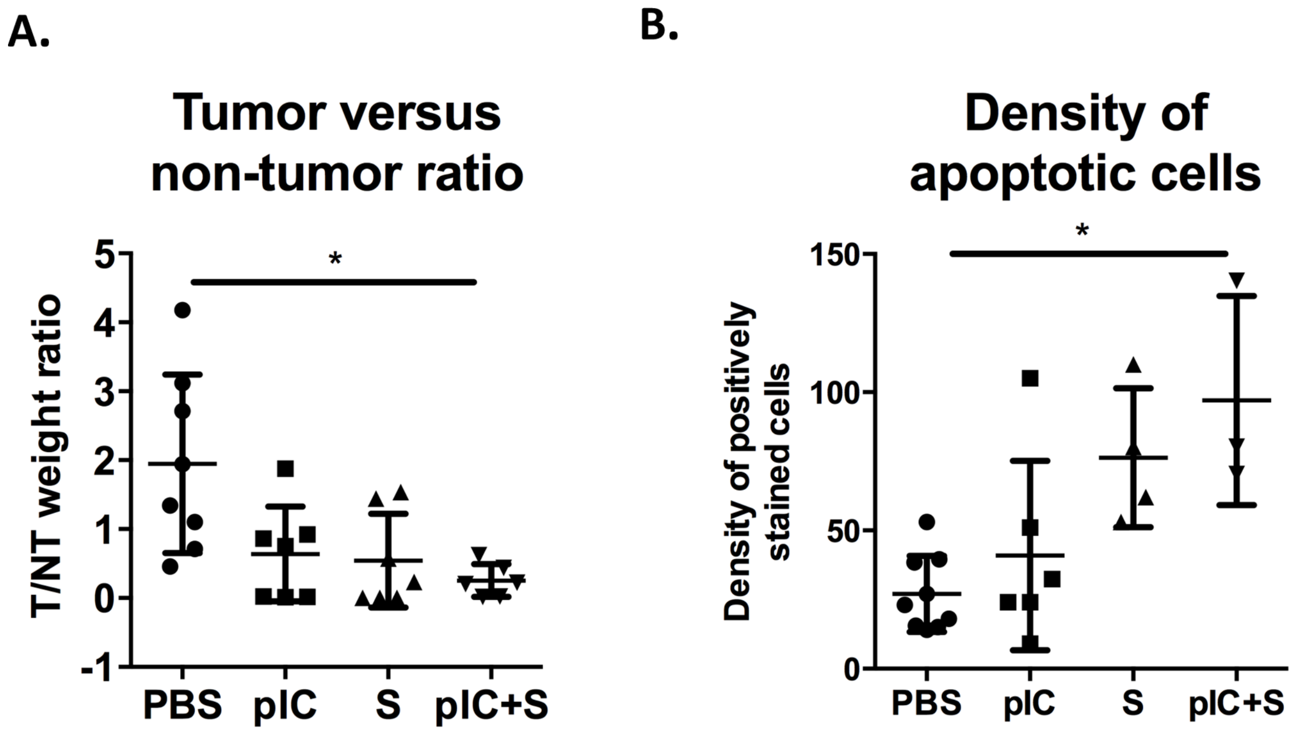

Supplementary Figure S2: Enhanced tumor control by combinatorial treatment in spontaneous model of liver tumors. C57BL/6 mice were induced to develop liver tumors spontaneously and treated as indicated in Figure 1D. **A.** Decreased mass ratio of liver tumor to healthy liver tissue (g) as harvested from mice treated with pIC+S at week 4. n = 6–9 from each treatment group. **B.** Increased density of apoptotic cells (TUNEL assay) was indicated by the increase in mean numbers of positively stained cells per tumor field from mice treated with pIC+S. n = 4–10. Mean and SD were shown. *$p < 0.05$, One-way ANOVA test with post Tukey's multiple comparisons test.

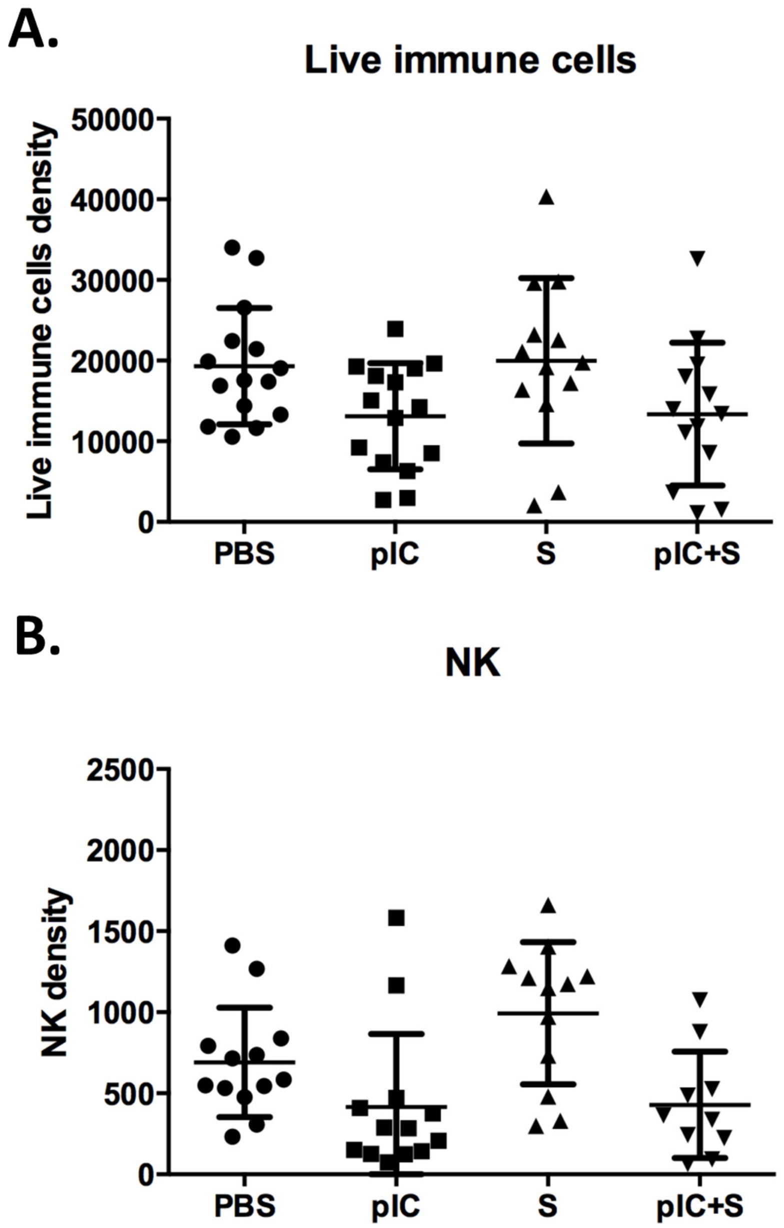

Supplementary Figure S3: No change in the density of TIL with combinatorial treatment in treated mice. C57BL/6 mice were transplanted with Hepa 1-6 cells in each flank and treated with either PBS, poly-ICLC (pIC), Sorafenib (S) or combination of both (pIC+S) as indicated in Figure 2C. Tumor-infiltrating leukocytes (TIL) were isolated and analysed using flow cytometry. Comparable densities of **A.** $CD45^{+}DAPI^{-}$ live immune cells **B.** $CD45^{+}NK1.1^{+}CD3^{-}DAPI^{-}$ NK cells. (*Continued*)

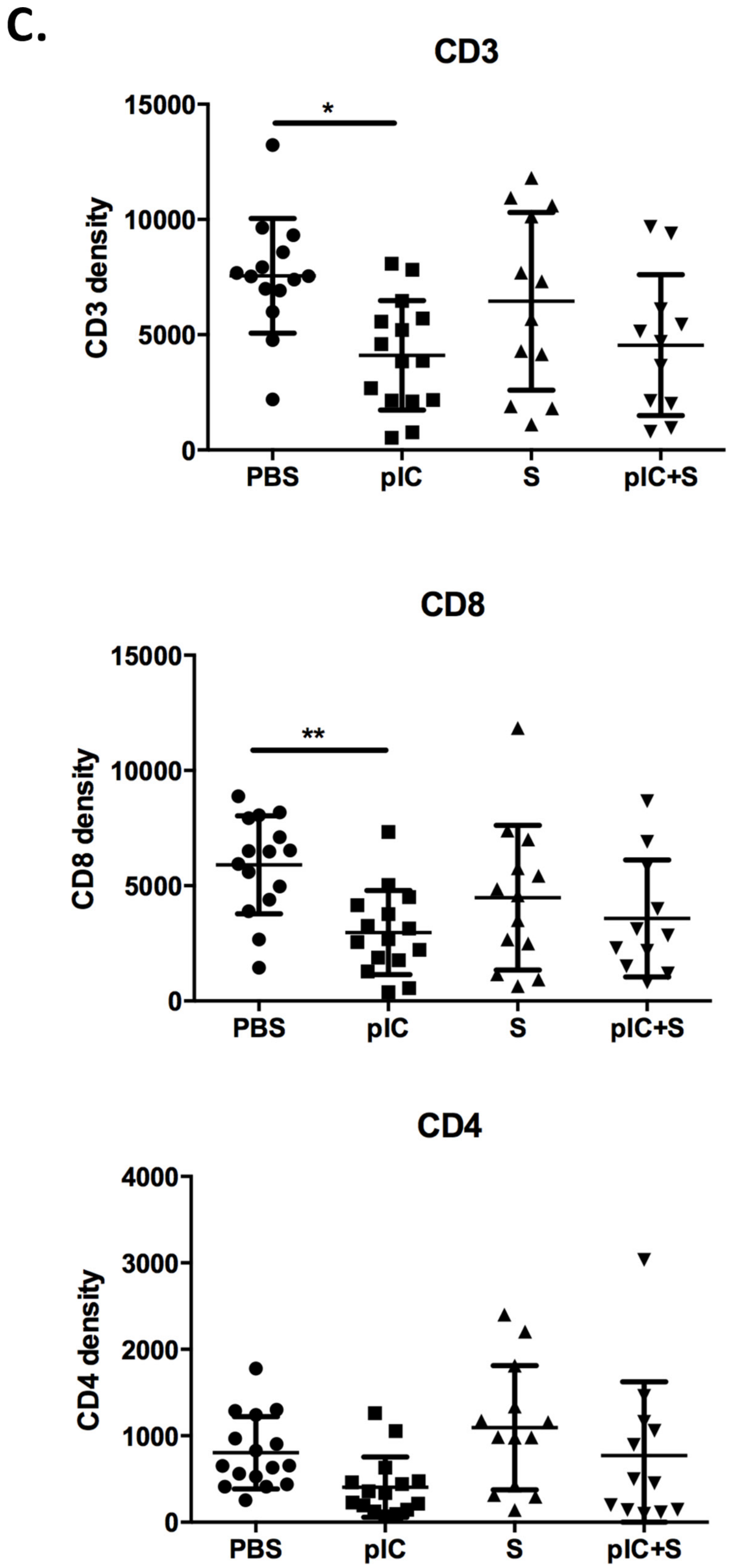

Supplementary Figure S3: (*Continued*) No change in the density of TIL with combinatorial treatment in treated mice. C. CD45$^+$DAPI$^-$CD3$^+$/CD4$^+$/CD8$^+$ T cells per mg of tumor weight from mice treated with pIC+S versus other treatments. n = 13–15 tumors. A–C. Mean and SD were shown. $^*p < 0.05$; $^{**}p < 0.01$, One-way ANOVA test with post Tukey's multiple comparisons test.

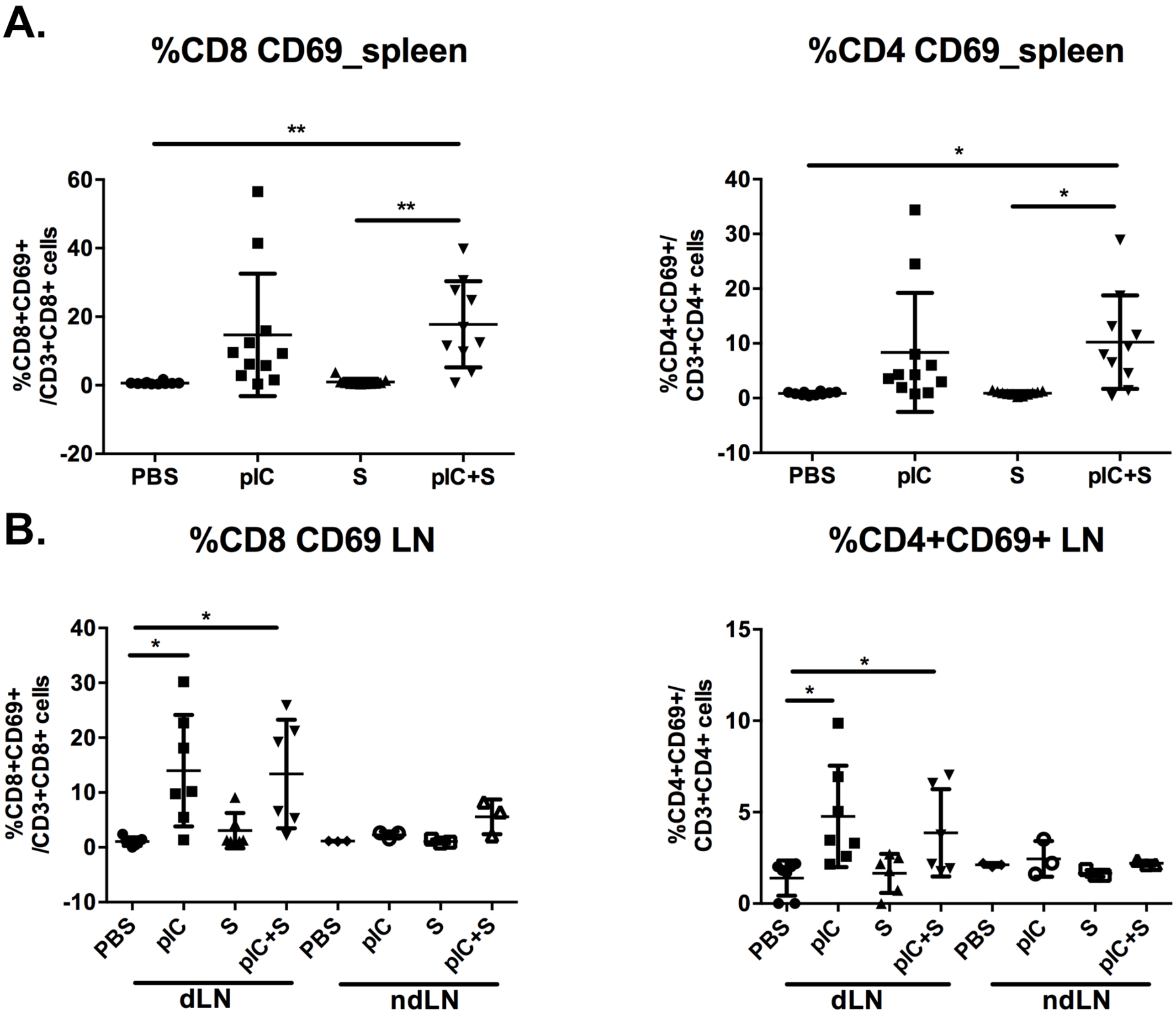

Supplementary Figure S4: Enhanced activation of $CD8^+$ and CD4+ T cells in the spleen and tumor-draining lymph-nodes with combinatorial treatment. C57BL/6 mice were transplanted with Hepa 1-6 cells in each flank and treated with either PBS, poly-ICLC(pIC), Sorafenib (S) or combination of both (pIC+S) as indicated in Figure 2C. Immune cells were isolated from spleen, $n = 10–11$; tumor draining inguinal and axillary lymph nodes (dLN), $n = 6–7$; and non-tumor-draining submandibular (ndLN) and parotid lymph nodes, $n = 3$ and analysed with flow cytometry. **A.** Increased percentage of $CD69^+$ on $CD8^+$ and $CD4^+$ T cells isolated from spleens in mice treated with pIC+S. **B.** Increased percentage of $CD69^+$ on $CD8^+$ and $CD4^+$ T cells isolated from tumor-draining inguinal and axillary lymph nodes relative to the non-draining submandibular and parotid lymph nodes in mice treated with pIC+S. A&B. Mean and SD were shown. $*p < 0.05$; $**p < 0.01$, One-way ANOVA test with post Tukey's multiple comparisons test.

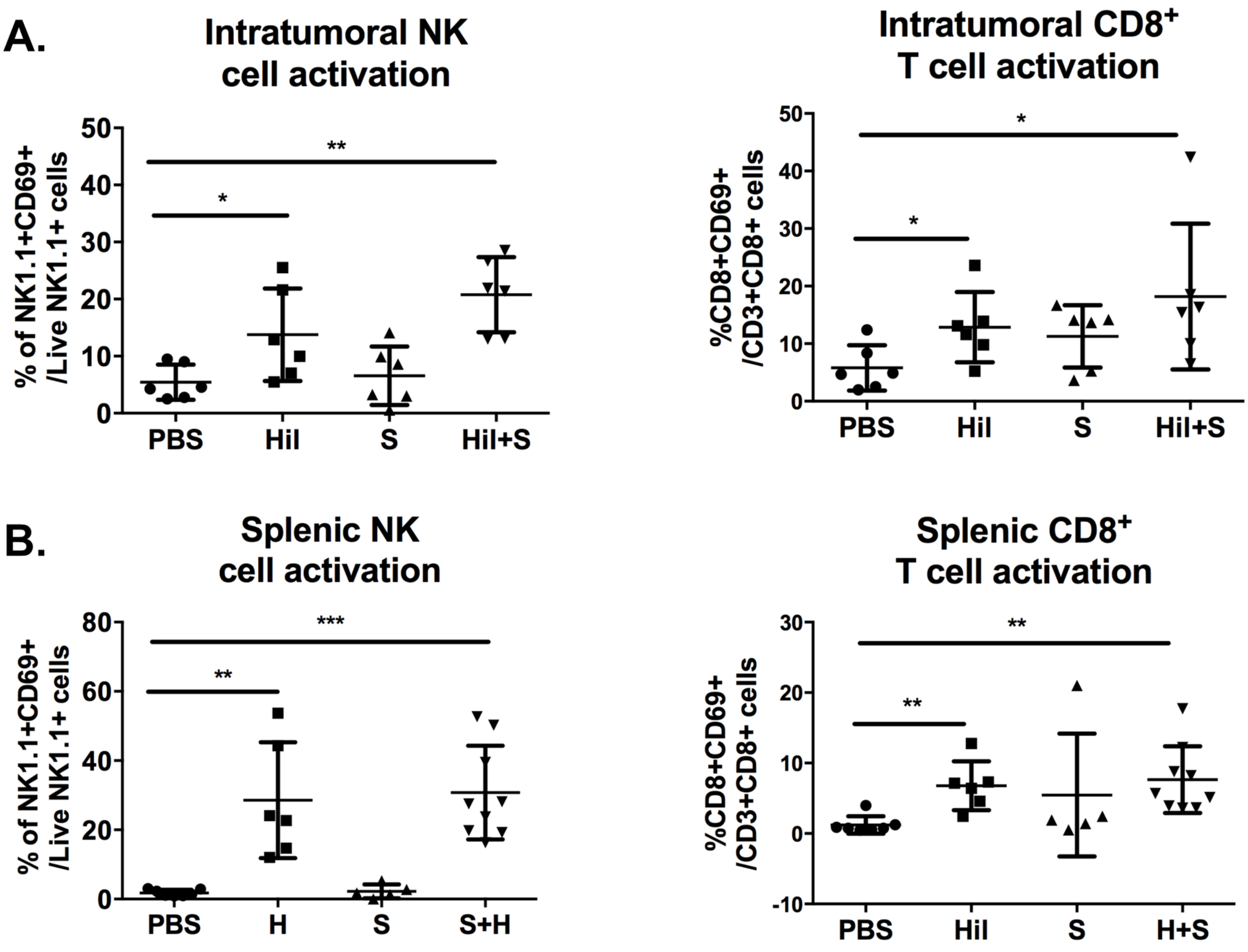

Supplementary Figure S5: Enhanced activation of NK cells and CD8+ T cells in the tumor and spleen with combinatorial treatment on spontaneous liver tumor model. C57BL/6 mice were induced to develop liver tumors spontaneously and treated as indicated in Figure 1D. Immune cells were isolated from tumor $n = 5–6$ or spleen, $n = 5–9$ and analysed with flow cytometry. **A.** Increased percentage of $CD69^+$ on NK cells and $CD8^+$ T cells isolated from tumors in mice treated with pIC+S. **B.** Increased percentage of $CD69^+$ on NK cells and $CD8^+$ T cells isolated from spleens in mice treated with pIC+S. A&B. Mean and SD were shown. $^*p < 0.05$; $^{**}p < 0.01$, $^{***}p < 0.0001$; One-way ANOVA test with post Tukey's multiple comparisons test.

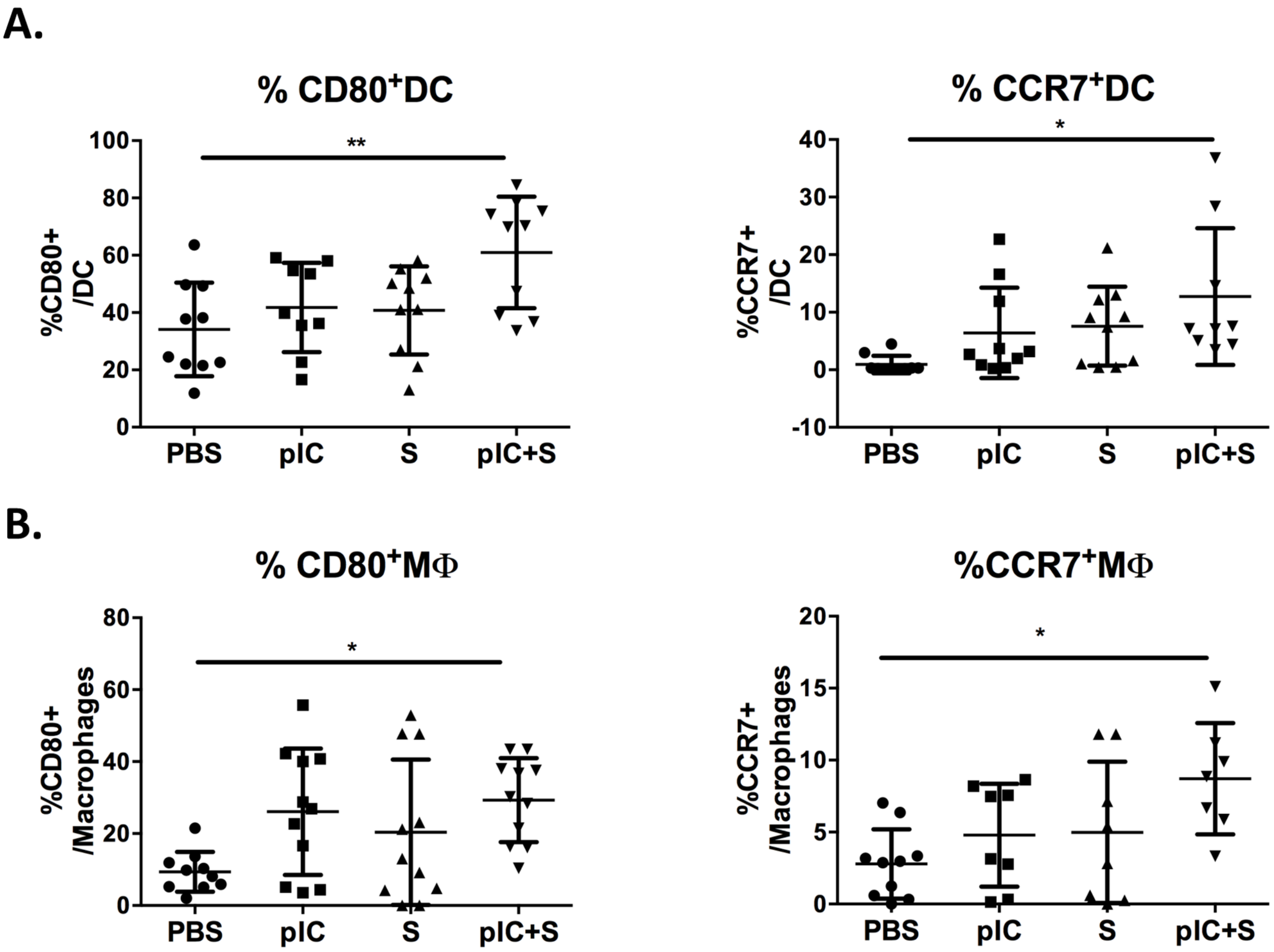

Supplementary Figure S6: Enhanced activation of dendritic cells and macrophages with combinatorial treatment. C57BL/6 mice were transplanted with Hepa 1-6 cells in each flank and treated with either PBS, poly-ICLC (pIC), Sorafenib (S) or combination of both (pIC+S) as indicated in Figure 2C. Tumor-infiltrating leukocytes (TIL) were isolated and analysed using flow cytometry. **A.** Increased percentage of $CD80^+$ and $CCR7^+$ dendritic cells (DC) in tumors from mice treated with pIC+S. **B.** Increased percentage of $CD80^+$ and $CCR7^+$ macrophages (MΦ) in tumors from mice treated with pIC+S. A&B. $n = 8–10$. Mean and SD were shown. *$p < 0.05$; **$p < 0.01$, One-way ANOVA test with post Tukey's multiple comparisons test.
